# Timescales and Mechanisms of Sigh-Like Bursting and Spiking in Models of Rhythmic Respiratory Neurons

**DOI:** 10.1186/s13408-017-0045-5

**Published:** 2017-06-06

**Authors:** Yangyang Wang, Jonathan E. Rubin

**Affiliations:** 1 Mathematical Biosciences Institute, Ohio State University, Jennings Hall 3rd Floor, 1735 Neil Ave., Columbus, 43210 USA; 2 Department of Mathematics, University of Pittsburgh, 301 Thackeray Hall, Pittsburgh, 15260 USA

**Keywords:** Multiple timescales, Bursting, Geometric singular perturbation theory, Respiratory neuron

## Abstract

Neural networks generate a variety of rhythmic activity patterns, often involving different timescales. One example arises in the respiratory network in the pre-Bötzinger complex of the mammalian brainstem, which can generate the eupneic rhythm associated with normal respiration as well as recurrent low-frequency, large-amplitude bursts associated with sighing. Two competing hypotheses have been proposed to explain sigh generation: the recruitment of a neuronal population distinct from the eupneic rhythm-generating subpopulation or the reconfiguration of activity within a single population. Here, we consider two recent computational models, one of which represents each of the hypotheses. We use methods of dynamical systems theory, such as fast-slow decomposition, averaging, and bifurcation analysis, to understand the multiple-timescale mechanisms underlying sigh generation in each model. In the course of our analysis, we discover that a third timescale is required to generate sighs in both models. Furthermore, we identify the similarities of the underlying mechanisms in the two models and the aspects in which they differ.

## Introduction

Many years of experimental work have elucidated a range of properties of the neuronal circuits involved in breathing. While there is clear evidence that one or more neuronal populations in the mammalian brain stem, including the well-studied pre-Bötzinger complex (pre-BötC) [1], are capable of generating respiratory-related rhythms, there is a lack of consensus about the transfer from rhythm generation to patterned motor output. While some have presented evidence that modulations of the interactions within a common rhythmogenic core can account for a variety of patterns of activity observed in recordings from respiratory pathways [2], others have argued that rhythm generation and pattern generation are distinct, with one or more pattern-generating respiratory populations shaping stereotyped baseline rhythms into different outputs [3, 4]. Recent computational work has shown, for example, that a distinct pattern generator is not necessary to explain the observation of mixed-mode oscillations composed of intermingled bursts and burstlets in respiratory neuron population readout nerves [5]. Nonetheless, further study is needed to help distinguish between these competing views.

One familiar example of a non-standard respiratory output pattern is the sigh. Sighs can be distinguished in neuronal activity; indeed, recordings of population activity from the ventral respiratory group (VRG) identified two distinct patterns of inspiratory activity under conditions of normal oxygenation: fictive eupnea (typical respiration) and low-frequency fictive sighs. As described by [6], fictive sighs occur periodically in the in vitro transverse medullary slice preparation from mice containing the pre-BötC within the VRG. Each fictive sigh consists of a biphasic activity burst that is larger in amplitude, longer in duration, and occurs at a lower frequency than eupneic bursts. Despite the experimental accessibility of sighs, the rhythmogenic mechanisms underlying sigh generation remain largely unknown [6, 7]. The biphasic aspect of the sigh, with an initial phase that is identical to a normal eupneic burst and a later high-amplitude phase, could result from the recruitment of a neuronal population distinct from the eupneic rhythm-generating circuit, or it could simply emerge due to the complex interplay of multiple-timescale processes within the core rhythm-generating circuit itself. The main goal of this paper is to use mathematical tools to elucidate the multiple-timescale mechanisms underlying sigh generation in two recent computational models [8, 9], one of which represents each of the competing hypotheses about pattern generation. In doing so, we will highlight the ways in which the dynamic mechanisms in the two models are in fact similar as well as ways that they can be distinguished in future experimental studies, to help determine whether or not separate pattern generating components complement rhythm generators in producing respiratory outputs.

**Fig. 1:**
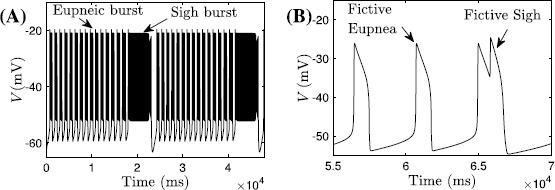
Sigh-like bursting and spiking solutions. Sigh-like bursting solution (**A**) and sigh-like spiking solution (**B**) from the models presented in [8] and [9], respectively. Patterns repeat periodically. In (**B**), we only plot a few of the many eupneic cycles occurring between sighs, to obtain better resolution in displaying the sigh

Example sigh patterns produced by the two models appear in Fig. 1. The model yielding the solution shown in Fig. 1A includes fast-spiking currents in addition to rhythmic burst generation, while the one associated with Fig. 1B does not, leading to the significant quantitative differences between their outputs. For convenience, we refer to such solutions as sigh-like bursting (SB, Fig. 1A) and sigh-like spiking (SS, Fig. 1B), respectively. Of course, the meaning of a ‘spike’ is quite different across the two models, representing a single action potential in the SB case and an entire active period in the SS model. Nonetheless, both feature high-amplitude, low-frequency, long-duration events emerging periodically on the top of higher-frequency baseline patterns. Indeed, a comparison of the patterns in Fig. 1 suggests that the fundamental dynamic mechanisms underlying both may be similar, analogously to the comparison between square-wave (or fold-homoclinic) bursting with spikes and relaxation oscillations without them. One of the contributions of our analysis will be to determine the extent to which this analogy holds, which will clarify the relation of these models for subsequent studies.

Experimental studies in rodent medullary slices containing the pre-BötC have identified two biophysical mechanisms that could potentially contribute to the generation of rhythmic bursting, one based on the persistent $\mathrm{Na}^{+}$ current ($I_{\mathrm{NaP}}$), and the other involving the voltage-gated $\mathrm{Ca}^{2+}$ current ($I_{\mathrm{Ca}}$) and the $Ca^{2+}$-activated nonspecific cation current ($I_{\mathrm{CAN}}$), activated by intracellular $\mathrm{Ca}^{2+}$, which may be accumulated from a variety of sources [10, 11]. Past computational work showed that the interactions of these burst mechanisms could yield a form of mixed bursting (MB) output with significant qualitative similarity to the SB pattern shown in Fig. 1A [12–14]. By applying methods of dynamical systems theory to a single-compartment model of a pre-BötC inspiratory neuron in [15], we explained in full detail how the MB solution results from these currents. In this paper, we generalize the analysis and methods for studying MB solutions in [15] to the more complicated model SB and SS models presented in Sect. 2 in order to uncover the mechanisms underlying the these patterns. Our approach is geometric and is based on studying reduced subsystems of an original model that evolve on particular timescales. It is not rigorous, in that we assume that there are abrupt transitions between timescales and we do not prove any results; moreover, we will sometimes make approximations, such as treating a nullcline that only weakly depends on a parameter as fixed under variations of that parameter. Nonetheless, this approach has a long history of providing powerful insights, for example, in work ensuing from the classical dissection of minimal bursting models by [16] and in the study of coupled neuronal oscillators and bursters (e.g, [17–20] and many others since). An important aspect of this dynamical systems approach is that it can decompose a solution pattern into a sequence of dynamic epochs evolving on different timescales and bifurcations of subsystems that underlie transitions between these epochs [21]. Thus, the approach distills out a set of key features that can be used to classify solutions and to objectively compare solutions that differ quantitatively and come from models that are superficially quite different, as in the famous classification of neuronal bursters [16, 22].

While the MB solution appears to involve at least three timescales based on its time course, we obtained a non-intuitive result in [15] that the core mechanism underlying the robust production of the MB pattern is in fact an interaction of only two timescales. In this paper, after presenting the SB and SS models in Sect. 2, we similarly analyze the timescale-interaction mechanisms that support SB solutions (in Sect. 3) and SS solutions (in Sect. 4). In both cases, we determine that timescales must remain separated into three distinct classes for the solutions of interest to arise. In Sect. 5, we compare these two sighing solutions to highlight the similarities and differences in the mechanisms and timescale interactions involved in producing them, and we conclude with a discussion in Sect. 6.

## Computational Models for Sighing

### Sigh-Like Bursting Model

We consider two recent models for neurons in the pre-BötC. Jasinski et al. [8] presented a relatively detailed model in the Hodgkin-Huxley framework for pre-BötC neurons and showed that a synaptically coupled population of these neurons, with heterogeneous parameter values, can generate SB solutions (Fig. 1A), whereas a single model pre-BötC neuron without synaptic inputs cannot produce a sighing rhythm. Beyond issues of synaptic coupling, the model by Jasinski et al. [8] is more complicated than the MB model [15] in two important ways. First, in addition to $I_{\mathrm{NaP}}$ and $I_{\mathrm{CAN}}$, the $\mathrm{Na}^{+}/\mathrm{K}^{+}$ pump plays an important role in the generation of activity patterns in this model. Furthermore, we have considered only one-directional coupling, from calcium concentration to the voltage dynamics, in [15], such that the MB model can be thought of as one oscillator forcing another (see also [23]). In the model developed by Jasinski et al., however, the membrane potential and the cytoplasmic $\mathrm{Ca}^{2+}$ concentration can each influence the evolution of the other.

To facilitate the identification of the essential mechanisms underlying the SB behavior and the assessment of how to group the timescales involved, we consider a synaptically self-coupled single-compartment model neuron based on the model presented in [8], which we refer to as the *Jasinski model*:

1a $$\begin{aligned} C\frac{dV}{dt} =&-I_{\mathrm{Na}}-I_{\mathrm{NaP}}-I_{\mathrm {K}}-I_{\mathrm{Ca}}-I_{\mathrm{CAN}}-I_{\mathrm{Pump}}-I_{\mathrm {L}}-I_{\mathrm{SynE}}, \end{aligned}$$

1b $$\begin{aligned} \frac{dy}{dt} =&\bigl(y_{\infty}(V)-y\bigr)/{\tau_{y}(V)}, \\ \ \ \ \ y =&\{m_{\mathrm{Na}}, h_{\mathrm{Na}}, m_{\mathrm{NaP}}, h_{\mathrm{NaP}}, m_{\mathrm{Ca}}, h_{\mathrm{Ca}}, m_{\mathrm{K}}\}, \end{aligned}$$

1c $$\begin{aligned} \frac{d\mathrm{Ca}_{\mathrm{tot}}}{dt} =&-\alpha_{\mathrm{Ca}} I_{\mathrm{Ca}}- \mathrm{Ca}_{\mathrm{i}}/\tau _{\mathrm{Ca}}, \end{aligned}$$

1d $$\begin{aligned} \frac{d\mathrm{Ca}_{\mathrm{i}}}{dt} =& -\alpha_{\mathrm{Ca}} I_{\mathrm{Ca}}- \mathrm{Ca}_{\mathrm{i}}/\tau _{\mathrm{Ca}}+ \mathrm{K}_{\mathrm {C}a}(J_{\mathrm{ER}_{\mathrm{IN}}}-J_{\mathrm{ER}_{\mathrm{OUT}}}), \end{aligned}$$

1e $$\begin{aligned} \frac{dl}{dt} =& AK_{\mathrm{d}}(1-l)-A\mathrm{Ca}_{\mathrm{i}} l, \end{aligned}$$

1f $$\begin{aligned} \frac{d\mathrm{Na}_{\mathrm{i}}}{dt} =& -\alpha_{\mathrm{Na}}(I_{\mathrm{Na}}+I_{\mathrm {NaP}}+I_{\mathrm{CAN}}+3I_{\mathrm{Pump}}), \end{aligned}$$

1g $$\begin{aligned} \frac{ds}{dt} =&\bigl((1-s)s_{\infty}(V)-s\bigr)/\tau_{s}(V). \end{aligned}$$

The neuronal membrane potential (*V*) is governed by a set of membrane ionic currents, as shown in (1a). *C* is neuronal membrane capacitance, which we set to $36~\mbox{pF}$, and *t* is time. The ionic currents in the model include fast $\mathrm{Na}^{+}$ current ($I_{\mathrm{Na}}$), persistent $\mathrm{Na}^{+}$ current ($I_{\mathrm{NaP}}$), delayed rectifier $\mathrm{K}^{+}$ current ($I_{\mathrm{K}}$), high-voltage-activated $\mathrm{Ca}^{2+}$ current ($I_{\mathrm{Ca}}$), $\mathrm{Ca}^{2+}$-activated nonspecific cation current ($I_{\mathrm{CAN}}$), $\mathrm{Na}^{+}/\mathrm{K}^{+}$ pump current ($I_{\mathrm{Pump}}$), leak current ($I_{\mathrm{L}}$) and excitatory synaptic current ($I_{\mathrm{SynE}}$), which combines self-coupling with tonic drive from respiratory feedback regions; see Table 1.

**Table 1:** **Ionic currents and channel reversal potentials for system (** **1a** **)–(** **1g** **)**

Currents (pA)	Reversal potentials (mV)
$I_{\mathrm{Na}}=\bar{g}_{\mathrm{Na}} m^{3}_{\mathrm{Na}} h_{\mathrm {Na}} (V-E_{\mathrm{Na}})$	$E_{\mathrm{Na}}= (R T/F) \ln(\mathrm{Na}_{\mathrm{o}}/\mathrm{Na}_{\mathrm{i}})$
$I_{\mathrm{NaP}}=\bar{g}_{\mathrm{NaP}} m_{\mathrm{NaP}} h_{\mathrm {NaP}} (V-E_{\mathrm{Na}})$	
$I_{\mathrm{K}}=\bar{g}_{\mathrm{K}} m^{4}_{\mathrm{K}} (V-E_{\mathrm {K}})$	$E_{\mathrm{K}}=(R T/F) \ln(\mathrm{K}_{\mathrm{o}}/\mathrm{K}_{\mathrm{i}})$
$I_{\mathrm{Ca}}=\bar{g}_{\mathrm{Ca}} m_{\mathrm{Ca}} h_{\mathrm{Ca}} (V-E_{\mathrm{Ca}})$	$E_{\mathrm{Ca}}= (R T/2F) \ln(\mathrm {Ca}_{\mathrm{o}}/\mathrm{Ca}_{\mathrm{i}})$
$I_{\mathrm{CAN}}=\bar{g}_{\mathrm{CAN}} m_{\mathrm{CAN}} (V-E_{\mathrm {CAN}})$	$E_{\mathrm{CAN}}=0$
$I_{\mathrm{Pump}}=R_{\mathrm{Pump}} (\varphi(\mathrm{Na}_{\mathrm{i}})-\varphi (\mathrm{Na}_{\mathrm {i}eq}))$, where $\varphi(x)=x^{3}/(x^{3}+K_{\mathrm{P}}^{3})$	
$I_{\mathrm{L}}= g_{\mathrm{L}} (V-E_{\mathrm{L}})$	$E_{\mathrm {L}}=-68$
$I_{\mathrm{SynE}}= (g_{\mathrm{SynE}} s+g_{\mathrm{tonic}}) (V-E_{\mathrm{SynE}})$	$E_{\mathrm{SynE}}=-10$

Activation (*m*) and inactivation (*h*) variables for most ionic channels are governed by Eq. (1b), where steady-state activation ($m_{\infty}$) and inactivation ($h_{\infty}$) functions and time constants are described as in Table 2. Notice from the bottom row in Table 2 that unlike the other currents, $I_{\mathrm{CAN}}$ activates instantaneously. This activation ($m_{\mathrm{CAN}}$) depends on the intracellular calcium concentration ($\mathrm{Ca}_{\mathrm{i}}$) and is voltage-independent. The parameters for these currents are specified in Table 3.

**Table 2:** **Functions associated with activation and inactivation variables for system (** **1a** **)–(** **1g** **). We use the variable** ***y*** **when an expression corresponds to a set of variables**

Gating variables	Steady-state activation and inactivation	Time constants
$m_{\mathrm{Na}}$	$y_{\infty}(V)= 1/(1+\exp (-(V-V_{y1/2})/k_{y}))$	$\tau_{y}(V)=\tau_{y\max}/\cosh (-(V-V_{\tau y1/2})/ k_{\tau y})$
$h_{\mathrm{Na}}$		
$m_{\mathrm{NaP}}$		
$h_{\mathrm{Na}}$		
$m_{\mathrm{Ca}}$		$\tau_{m_{\mathrm{Ca}}}=0.5~\mbox{ms}$
$h_{\mathrm{Ca}}$		$\tau_{h_{\mathrm{Ca}}}=18~\mbox{ms}$
$m_{\mathrm{K}}$	${m_{\mathrm{K}}}_{\infty}= \alpha _{\infty}/(\alpha_{\infty}+\beta_{\infty})$	$\tau_{m_{\mathrm {K}}}=1/(\alpha_{\infty}+\beta_{\infty})$
	$\alpha_{\infty}= A_{\alpha }\cdot(V+B_{\alpha})/(1-\exp(-(V+B_{\alpha})/k_{\alpha}))$, $\beta _{\infty}=A_{\beta}\cdot\exp(-(V+B_{\beta})/k_{\beta})$	
$m_{\mathrm{CAN}}$	$m_{\mathrm{CAN}}=1/(1+(K_{\mathrm {CAN}}/\mathrm{Ca}_{\mathrm{i}})^{n})$

**Table 3:** **Parameter values for system (** **1a** **)–(** **1g** **)**

Current	Parameters
Fast ${\mathrm{Na}}^{+}$ ($I_{\mathrm{Na}}$)	$\bar{g}_{\mathrm {Na}}=150~\mbox{nS}$, $RT/F=26.54~\mbox{mV}$, $\mathrm{Na}_{\mathrm{o}}=120~\mbox{mM}$
	$V_{m1/2} = -43.8~\mbox{mV}$, $k_{m} = 6~\mbox{mV}$, $\tau_{m \max} = 0.25~\mbox{ms}$, $V_{\tau m1/2} = -43.8~\mbox{mV}$, $k_{\tau m} = 14~\mbox{mV}$
	$V_{h1/2} = -67.5~\mbox{mV}$, $k_{h} = -10.8~\mbox{mV}$, $\tau_{h \max} = 8.46~\mbox{ms}$, $V_{\tau h1/2} = -67.5~\mbox{mV}$, $k_{\tau h} = 12.8~\mbox{mV}$
Persistent ${\mathrm{Na}}^{+}$ ($I_{\mathrm{NaP}}$)	$\bar{g}_{\mathrm{NaP}} = 0~\mbox{nS}$
	$V_{m1/2} = -47.1~\mbox{mV}$, $k_{m} = 3.1~\mbox{mV}$, $\tau_{m \max} = 1~\mbox{ms}$, $V_{\tau m1/2} = -47.1~\mbox{mV}$, $k_{\tau m} = 6.2~\mbox{mV}$
	$V_{h1/2} = -60~\mbox{mV}$, $k_{h} = -9~\mbox{mV}$, $\tau_{h \max} = 5\text{,}000~\mbox{ms}$, $V_{\tau h1/2} = -60~\mbox{mV}$, $k_{\tau h} = 9~\mbox{mV}$
	In the case of non-inactivating $I_{\mathrm{NaP}}$, *h* = constant = 0.4
$\mathrm {K}^{+}$ delayed rectifier ($I_{\mathrm {K}}$)	$\bar{g}_{\mathrm {K}} = 160~\mbox{nS}$, $\mathrm{K}_{\mathrm{o}}=4~\mbox{mM}$, $\mathrm{K}_{\mathrm{i}}=140~\mbox{mM}$
	$A_{\alpha}= 0.01$, $B_{\alpha}= 44~\mbox{mV}$, $k_{\alpha}= 5~\mbox{mV}$, $A_{\beta}= 0.17$, $B_{\beta}= 49~\mbox{mV}$, $k_{\beta}= 40~\mbox{mV}$
${\mathrm{Ca}}^{2+}$ ($I_{\mathrm{Ca}}$)	$\bar{g}_{\mathrm{Ca}} = 0.00065~\mbox{nS}$, $\mathrm{Ca}_{\mathrm{o}}=4~\mbox{mM}$
	$V_{m1/2} = -27.5~\mbox{mV}$, $k_{m} = 5.7~\mbox{mV}$
	$V_{h 1/2} = -52.4~\mbox{mV}$, $k_{h} = -5.2~\mbox{mV}$
${\mathrm{Ca}}^{2+}$-activated nonspecific ($I_{\mathrm{CAN}}$)	$\bar {g}_{\mathrm{CAN}} = 3~\mbox{nS}$, $K_{\mathrm{CAN}} = 0.00074~\mbox{mM}$, *n* = 0.97
${\mathrm{Na}}^{+}/{\mathrm{K}}^{+} {\mathrm{pump}} (I_{\mathrm{Pump}})$	$R_{\mathrm{Pump}} = 200~\mbox{pA}$, ${\mathrm{Na}}_{\mathrm{ieq}}=15~\mbox{mM}$, $K_{p} = 15~\mbox{mM}$
Leakage ($I_{\mathrm {L}}$)	$g_{\mathrm {L}} = 2.5~\mbox{nS}$
Excitatory synaptic ($I_{\mathrm{SynE}}$)	$g_{\mathrm{SynE}} = 20~\mbox{nS}$, $g_{\mathrm{tonic}}=0.78~\mbox{nS}$

The dynamics of the total intracellular $\mathrm{Ca}^{2+}$ concentration within the cell ($\mathrm{Ca}_{\mathrm{tot}}$) and intracellular concentration of free $\mathrm{Ca}^{2+}$ ($\mathrm{Ca}_{\mathrm{i}}$) are described by (1c) and (1d), respectively, and these are intimately linked with *l*, the fraction of $\mathrm{IP}_{3}$ receptors that are not inactivated by calcium, which is governed by (1e). Calcium dynamics is influenced by voltage through the first term in the right-hand sides of (1c) and (1d), $-\alpha_{\mathrm{Ca}} I_{\mathrm{Ca}}$, which represents $\mathrm{Ca}^{2+}$ influx from the extracellular space through voltage-gated $\mathrm{Ca}^{2+}$ channels. In (1d), $J_{\mathrm{ERin}}$ represents the flux of $\mathrm{Ca}^{2+}$ per unit volume from the endoplasmic reticulum (ER) into the cytoplasm, which depends on *l*, and $J_{\mathrm{ERout}}$ represents the flux of $\mathrm{Ca}^{2+}$ per unit volume from the cytoplasm into the ER. These two fluxes are modeled by (2a)–(2c):

2a $$\begin{aligned} J_{\mathrm{ER}_{\mathrm{IN}}} =& \biggl(L_{\mathrm{IP}_{3}}+P_{\mathrm{IP}_{3}} \biggl[ \frac {[\mathrm{IP}_{3}]\mathrm{Ca}_{\mathrm{i}} l}{([\mathrm{IP}_{3}]+K_{I})(\mathrm {Ca}_{\mathrm{i}}+K_{a})} \biggr]^{3} \biggr) (\mathrm{Ca}_{\mathrm{ER}}- \mathrm{Ca}_{\mathrm{i}}), \end{aligned}$$

2b $$\begin{aligned} J_{\mathrm{ER}_{\mathrm{OUT}}} =& V_{\mathrm{SERCA}}\frac{\mathrm{Ca}_{i}^{2}}{ K^{2}_{\mathrm{SERCA}}+\mathrm{Ca}_{\mathrm{i}}^{2}}, \end{aligned}$$

2c $$\begin{aligned} \mathrm{Ca}_{\mathrm{ER}} =& \frac{\mathrm{Ca}_{\mathrm{tot}}-\mathrm{Ca}_{\mathrm{i}}}{\sigma} . \end{aligned}$$

The values of parameters associated with the equations for $\mathrm{Ca}_{\mathrm{tot}}$, $\mathrm{Ca}_{\mathrm{i}}$ and *l* are given by $\alpha_{\mathrm {Ca}}=2.5\times 10^{-5}~\mbox{mM}/\mbox{fC}$, $\tau_{\mathrm{Ca}}=500~\mbox{ms}$, $\mathrm{K}_{\mathrm{Ca}}=2.5\times 10^{-5}$, $L_{\mathrm{IP}_{3}\mathrm{R}}=0.37/\mbox{ms}$, $P_{\mathrm{IP}_{3}}=31\text{,}000/\mbox{ms}$, $[\mathrm{IP}_{3}]=1.5 \times10^{-3}~\mbox{mM}$, $K_{I}=10^{-3}~\mbox{mM}$, $K_{a}=0.4\times 10^{-3}~\mbox{mM}$, $V_{\mathrm{SERCA}}=0.4~\mbox{mM}/\mbox{ms}$, $K_{\mathrm{SERCA}}=0.2\times10^{-3}~\mbox{mM}$, $\sigma=0.185$, $A=0.1~\mbox{mM}/\mbox{ms}$ and $K_{\mathrm{d}}=0.4\times10^{-3}~\mbox{mM}$ [13]. Additional description of model components has been given previously [13, 14]. For convenience, we henceforth omit units from the model parameters and variables.

### Sigh-Like Spiking Model

Toporikova et al. [9] recently designed a computational model for inspiratory pre-BötC neurons, based on an earlier model [13] that includes two different bursting mechanisms depending on $I_{\mathrm{NaP}}$ and intracellular $\mathrm{Ca}^{2+}$, respectively. In contrast to the Jasinski model, this model does not include fast-spiking components (i.e., $I_{\mathrm{Na}}$ and $I_{\mathrm{K}}$) such that instead of generating SB solutions, it produces SS patterns. Under different parameter choices emphasizing distinct burst-generating mechanisms, this model generates oscillations following mainly the kinetics of $I_{\mathrm{NaP}}$ or an even lower-frequency sigh-like rhythm resulting mainly from slow $\mathrm{Ca}^{2+}$ oscillations. Following the terminology used in [9], we can instantiate two copies of the model, one with each type of parameter set, and refer to the model equipped with parameters that support fictive sigh activity as the sigh compartment and the model with parameters that support eupneic activity as the eupnea compartment.

**Fig. 2:**
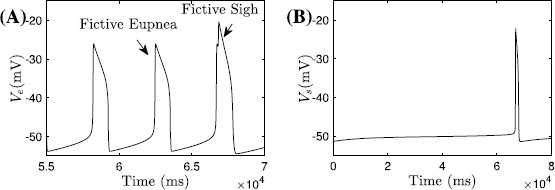
Fictive eupnea and sighs. The model in [9] generates both fictive eupnea (lower amplitude, higher frequency) and fictive sighs (lower frequency, higher amplitude). (**A**) Voltage trace ($V_{e}$) for the eupnea compartment with unidirectional input from the sigh compartment. (**B**) Voltage trace for the isolated sigh compartment, as specified in Eqs. (3a)–(3e) and (4a)–(4j); here we use $V_{s}$ in place of *V* to contrast with the voltage $V_{e}$ in (**A**). Note the difference in timescales between the two panels

With an inhibitory synapse from the eupnea compartment to the sigh compartment and an excitatory synapse from sigh to eupnea, the output of the coupled model is the SS pattern (see Fig. 1B) composed of a large spike emerging periodically on the top of regular spikes, analogous to the long bursts separated by short bursts in the SB solution, which is consistent with experimental data obtained *in vitro* [6, 7, 24]. In fact, the role of the coupling between compartments is simply to coordinate the timing of the eupneic and sigh-like spikes. For example, the oscillation pattern occurring after removal of the coupling from the eupnea compartment to the sigh compartment appears in Fig. 2A, while the spike of the sigh compartment alone, without input, is shown on a different timescale in Fig. 2B. Furthermore, the generation of eupneic spikes can be understood in the same way as the regular bursts in the SB solution, which we consider in Sect. 3.1.1. Hence we focus entirely on the sigh compartment on its own in our analysis in Sect. 4.

For simplicity, we will still denote the solution shown in Fig. 2B as the SS solution and we refer to the sigh compartment model as the *Toporikova model*, described by the following equations:

3a $$\begin{aligned} C_{m}\frac{dV}{dt} =&-I_{\mathrm{NaP}}-I_{\mathrm{leak}}-I_{\mathrm {CAN}}-I_{\mathrm{Ca}}-I_{h}, \end{aligned}$$

3b $$\begin{aligned} \frac{dh}{dt} =&\bigl(h_{\infty}(V)-h\bigr)/{\tau_{h}(V)}, \end{aligned}$$

3c $$\begin{aligned} \frac{d\mathrm{Ca}_{\mathrm{tot}}}{dt} =&\frac{\mathrm{K}_{\mathrm{Ca}}}{\lambda}(J_{\mathrm{PM}_{\mathrm{IN}}}-J_{\mathrm{PM}_{\mathrm{OUT}}}), \end{aligned}$$

3d $$\begin{aligned} \frac{d\mathrm{Ca}_{\mathrm{i}}}{dt} =& \frac{\mathrm{K}_{\mathrm{Ca}}}{\lambda}(J_{\mathrm{PM}_{\mathrm{IN}}}-J_{\mathrm{PM}_{\mathrm{OUT}}})+ \mathrm{K}_{\mathrm{Ca}}(J_{\mathrm{ER}_{\mathrm{IN}}}-J_{\mathrm{ER}_{\mathrm{OUT}}}), \end{aligned}$$

3e $$\begin{aligned} \frac{dl}{dt} =& AK_{\mathrm{d}}(1-l)-A \mathrm{Ca}_{\mathrm{i}} l , \end{aligned}$$

with

4a $$\begin{aligned} I_{\mathrm{NaP}} =& g_{\mathrm{NaP}}m_{\infty}h(V-V_{\mathrm{NaP}}), \end{aligned}$$

4b $$\begin{aligned} I_{\mathrm{leak}} =& g_{\mathrm {K}}(V-V_{\mathrm{K}}), \end{aligned}$$

4c $$\begin{aligned} I_{\mathrm{CAN}} =& g_{\mathrm{CAN}}\mathrm{CAN}_{\infty}(V-V_{\mathrm {NaP}}), \end{aligned}$$

4d $$\begin{aligned} \mathrm{CAN}_{\infty} =&1/(1+K_{\mathrm{CAN}}/\mathrm{Ca}_{\mathrm{i}}), \end{aligned}$$

4e $$\begin{aligned} I_{\mathrm{Ca}} =& g_{\mathrm{Ca}}m_{\infty}(V-V_{\mathrm{Ca}}), \end{aligned}$$

4f $$\begin{aligned} I_{h} =&g_{h}n_{\infty}(V-V_{H}), \end{aligned}$$

4g $$\begin{aligned} x_{\infty}(V) =& 1/\bigl(1+\exp\bigl((V-V_{x})/S_{x} \bigr)\bigr),\quad x\in\{m, n, h\} , \end{aligned}$$

4h $$\begin{aligned} \tau_{h}(V) =&\bar{\tau}_{h}/{\cosh}\bigl((V-V_{h})/2S_{h} \bigr), \end{aligned}$$

4i $$\begin{aligned} J_{\mathrm{PM}_{\mathrm{IN}}} =&-\alpha_{\mathrm{Ca}} I_{\mathrm{Ca}}, \end{aligned}$$

4j $$\begin{aligned} J_{\mathrm{PM}_{\mathrm{OUT}}} =&V_{\mathrm{PMCA}}\mathrm{Ca}_{\mathrm{i}}^{2}/ \bigl(K_{\mathrm{PMCA}}^{2}- \mathrm{Ca}_{\mathrm{i}}^{2} \bigr) . \end{aligned}$$

In this system, the terms $J_{\mathrm{ER}_{\mathrm{IN}}}$ and $J_{\mathrm{ER}_{\mathrm{OUT}}}$ appearing in the $\mathrm{Ca}_{\mathrm{i}}$ Eq. (3d) are the same as those used in the Jasinski model as given by Eqs. (2a)–(2c). Parameters related to them have been specified in Sect. 2.1, except $[\mathrm{IP}_{3}]=1~\mu \mbox{M}$. In Eqs. (3c)–(3d), *λ* is the ratio of ER to plasma membrane surfaces, which we set to 0.1. $C_{m}$, the neuronal membrane capacitance, is $21~\mbox{pF}$. Other parameter values related to currents and corresponding units for the Toporikova model are given in Table 4.

**Table 4:** **The values of the parameters in the sigh compartment model given by Eqs. (** **3a** **)–(** **3e** **) and (** **4a** **)–(** **4j** **)**

Current	Parameters
$I_{\mathrm{NaP}}$	$g_{\mathrm{NaP}} = 1.3~\mbox{nS}$, $V_{h}=-48~\mbox{mV}$, $V_{m}=-40~\mbox{mV}$, $S_{h}=5~\mbox{mV}$, $S_{m}=-6~\mbox{mV}$, $\bar{\tau}_{h}=10\text{,}000~\mbox{ms}$, $V_{\mathrm{NaP}}=50~\mbox{mV}$
$I_{\mathrm{leak}}$	$g_{K} = 2.7~\mbox{nS}$, $V_{\mathrm{K}}=-60~\mbox{mV}$
$I_{\mathrm{Ca}}$	$g_{\mathrm{Ca}} = 0.02~\mbox{nS}$, $V_{\mathrm {Ca}}=150~\mbox{mV}$, $\alpha_{\mathrm{Ca}}=0.055$, $V_{\mathrm{PMCA}}=2$, $K_{\mathrm{PMCA}}=0.3$
$I_{\mathrm{CAN}}$	$g_{\mathrm{CAN}} = 1.5~\mbox{nS}$, $K_{\mathrm {CAN}} = 0.00074~\mbox{mM}$
$I_{\mathrm{h}}$	$g_{\mathrm{h}} = 2~\mbox{nS}$, $S_{n}=8~\mbox{mV}$, $V_{n}=-70~\mbox{mV}$, $V_{H}=30~\mbox{mV}$

## Sigh-Like Bursting in a Self-Coupled Pre-BötC Neuron

At the single-neuron level, with the self-coupling removed by setting $g_{\mathrm{SynE}}=0$, model (1a)–(1g) can produce different intrinsic bursting patterns, depending on chosen parameter values. One type of bursting is based on the slow voltage-dependent inactivation of $I_{\mathrm{NaP}}$ (as represented by the $h_{\mathrm{NaP}}$ variable in the equation for $I_{\mathrm{NaP}}$ in Table 1), whereas another type relies on intracellular $\mathrm{Ca}^{2+}$ and depends on $I_{\mathrm{CAN}}$. There are several distinct burst-terminating mechanisms that can contribute, based, respectively, on the slow inactivation of $I_{\mathrm{NaP}}$, the activity-dependent accumulation of $\mathrm{Na}^{+}$ followed by the action of the $[\mathrm{Na}^{+}]_{\mathrm{in}}$-activated $I_{\mathrm{Pump}}$, and the $\mathrm{Ca}^{2+}$-dependent inactivation of $\mathrm{IP}_{3}$ receptors.

With synaptic coupling between two or more neurons (i.e., $g_{\mathrm{SynE}}>0$), the coupled cells are able to generate SB solutions. We consider the special case of a single self-coupled cell, given by model (1a)–(1g), as a reduction of a coupled network. Similarly to findings in [8], numerical simulations of (1a)–(1g) indicate that assigning $\bar{g}_{\mathrm{Ca}}=0$ or $\bar{g}_{\mathrm{CAN}}=0$ does not affect regular bursting, but fully removes the sigh-like oscillations and hence the SB pattern. These effects suggest that the generation of sigh-like bursts in this model is $I_{\mathrm {Ca}}/I_{\mathrm{CAN}}$-dependent, while regular bursting is independent of $I_{\mathrm{CAN}}$. Furthermore, setting $I_{\mathrm {Pump}}=0$ eliminates both regular bursting and sigh-like oscillations, implying that the $\mathrm{Na}^{+}/\mathrm{K}^{+}$ pump is also critically involved. On the other hand, simulations with $\bar{g}_{\mathrm{NaP}}=0$ show that the full SB solution survives intact without the need for $I_{\mathrm{NaP}}$.

**Fig. 3:**
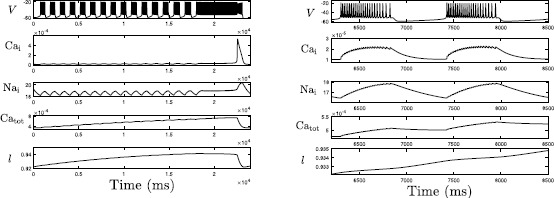
Simulation of rhythmic sigh-like bursting in the Jasinski model. Simulation of rhythmic sigh-like bursting in the Jasinski model (1a)–(1g). *From top to bottom*: Time courses of membrane potential, calcium, sodium, total intracellular calcium concentration within the cell ($\mathrm{Ca}_{\mathrm{tot}}$) and $\mathrm{IP}_{3}$ channel gating variable *l*. This SB pattern repeats periodically. The *right panel* provides a zoomed view showing the details of a regular small burst, where the *dashed green and blue lines* denote the end of a burst active phase and the beginning of the next such phase, respectively. Note the difference in time labels between the left and right panels

The left panel of Fig. 3 demonstrates the evolution of several variables, $\mathrm{Ca}_{\mathrm{i}}$, $\mathrm{Na}_{\mathrm{i}}$, $\mathrm{Ca}_{\mathrm{tot}}$ and *l*, during an SB cycle. Note that there exist low-amplitude $\mathrm{Ca}_{\mathrm{i}}$ and $\mathrm{Na}_{\mathrm{i}}$ transients during regular bursts, eventually followed by an abrupt increase of intracellular $\mathrm{Ca}^{2+}$, which $\mathrm{Na}_{\mathrm{i}}$ appears to follow more slowly. Meanwhile, $\mathrm{Ca}_{\mathrm{tot}}$ and *l*, which only interact directly with each other and $\mathrm{Ca}_{\mathrm{i}}$, both accumulate until $\mathrm{Ca}_{\mathrm{i}}$ jumps up and then start decreasing. The right panel of Fig. 3 shows in a magnified view of a regular burst that $\mathrm{Ca}_{\mathrm{i}}$, $\mathrm{Na}_{\mathrm{i}}$ and $\mathrm{Ca}_{\mathrm{tot}}$ engage in small oscillations during the spiking phase, consistent with the fact that these variables receive input from *V*; they tend to increase throughout this phase and to decrease while *V* is not spiking.

We aim to understand the mechanisms underlying this SB solution and to elucidate the timescales that are needed to produce it. The bidirectional coupling between membrane potential and cytoplasmic $\mathrm{Ca}^{2+}$ concentration, as well as the high-dimensionality of model (1a)–(1g), are complications not present in previous related analyses [15, 23]. To achieve these goals, we will nondimensionalize Eqs. (1a)–(1g) to reveal the presence of different timescales; determine how to group timescales that are present; implement geometric singular perturbation theory (GSPT) to set up reduced systems based on separation of timescales; and use the reduced systems to explain the mechanisms underlying the dynamics of the SB solutions. In contrast to the related MB model [15], it will turn out that use of the averaging method will play a role in the analysis. By uncovering the mechanisms underlying the SB solution in the Jasinski model, we will conclude that, unlike the case with the MB solution studied previously, a third timescale actually is required to generate SB solutions in this model.

### Analysis of Sigh-Like Bursting

Since the SB solution dynamics in the Jasinski model persists without $I_{\mathrm{NaP}}$, we can reduce (1a)–(1g) to an 11-dimensional system by removing $m_{\mathrm{NaP}}$ and $h_{\mathrm{NaP}}$ and setting $I_{\mathrm{NaP}}=0$ in Eq. (1a). Henceforth, we still refer to the new lower-dimensional model as the Jasinski model. The fact that regular bursting depends on $I_{\mathrm{Pump}}$ but not $I_{\mathrm{CAN}}$ and $I_{\mathrm{Ca}}$ suggests that we can decouple the $I_{\mathrm{Pump}}$-based burster from the calcium dynamics during the regular bursting phase of the SB solution. Hence, we can think of the Jasinski model as consisting of two subsystems: $(V, y, \mathrm{Na}_{\mathrm{i}}, s)$ (denoted as the *voltage compartment*) as a potentially bursting subsystem based on $I_{\mathrm{Pump}}$, and $(\mathrm{Ca}_{\mathrm{i}}, \mathrm{Ca}_{\mathrm{tot}}, l) $ (denoted as the *calcium compartment*) as a potentially oscillating subsystem. We first investigate the $I_{\mathrm{Pump}}$-based mechanism underlying the regular burst generated by the voltage compartment through a bifurcation analysis and then study the effect of the calcium compartment on the resulting bifurcation structures to understand how the transition from regular bursts to the long burst happens. Of course, we will have to take into account the coupling from the voltage compartment to the calcium compartment to complete the analysis.

Our methods for analyzing the model will depend heavily on exploiting the presence of different time scales. As a first step, it is helpful to rescale the variables so that the important timescales can be explicitly identified and used to group variables into timescale classes that are different from the voltage and calcium compartment groupings, which are based on coupling structure and biology. To this end, we define new dimensionless variables $(v, ca_{i}, c_{\mathrm{tot}}, na_{i}, \tau)$, and voltage, calcium, sodium and timescales $Q_{v}$, $Q_{c}$, $Q_{na}$, and $Q_{t}$, respectively, such that

$$\begin{aligned} V =&Q_{v}\cdot v,\qquad \mathrm{Ca}_{\mathrm{i}}=Q_{c} \cdot ca_{i},\qquad \mathrm {Ca}_{\mathrm{tot}}=Q_{c}\cdot c_{\mathrm{tot}}, \\ \mathrm{Na}_{\mathrm{i}} =&Q_{na}\cdot na_{i},\qquad t=Q_{t}\cdot\tau. \end{aligned}$$

Note that *y*, *s* and *l* are already dimensionless in (1a)–(1g).

Details of the nondimensionalization procedure, including the determination of appropriate values for $Q_{v}$, $Q_{c}$, $Q_{na}$ and $Q_{t}$, are given in Appendix 1. From this process, we obtain a dimensionless system of the form

5a $$\begin{aligned} R_{v}\frac{dv}{dt} =& f(v,y,s,ca_{i},na_{i}), \end{aligned}$$

5b $$\begin{aligned} R_{y}\frac{dy}{d\tau} =& H(v,y), \end{aligned}$$

5c $$\begin{aligned} R_{c_{\mathrm{tot}}}\frac{d c_{\mathrm{tot}}}{d\tau} =& h_{1}(v,ca_{i}), \end{aligned}$$

5d $$\begin{aligned} R_{ca_{i}}\frac{d ca_{i}}{d\tau} =& g_{1}(v,ca_{i},c_{\mathrm{tot}},l), \end{aligned}$$

5e $$\begin{aligned} R_{l}\frac{dl}{d\tau} =& h_{2}(ca_{i},l), \end{aligned}$$

5f $$\begin{aligned} R_{na_{i}}\frac{d na_{i}}{d\tau} =& g_{2}(v,na_{i},ca_{i}), \end{aligned}$$

5g $$\begin{aligned} R_{s}\frac{ds}{d\tau} =& S(v,s) , \end{aligned}$$

with coefficients of derivatives on the left-hand sides as well as functions on the right-hand sides specified in Eqs. (14a)-(14g), and timescales for all variables shown in Table 5, both of which appear in Appendix 1. While *v*, gating variables $m_{\mathrm{Na}}$, $h_{\mathrm{Na}}$, $m_{\mathrm{Ca}}$, $h_{\mathrm{Ca}}$, $m_{\mathrm{K}}$, and *s* do not operate on exactly the same timescale quantitatively, it is clear that they are all relatively faster than the other variables. Hence we choose to group all of them as fast variables, to consider $na_{i}$ and $ca_{i}$ as slow, and to classify *l* and $c_{\mathrm{tot}}$ as evolving on a superslow timescale. For simplicity, we abuse notation to now let $y\in\mathbb{R}^{6}$ denote all the fast gating variables along with *s*. For each group of variables we can define a corresponding subsystem of equations with slower variables kept as parameters, as we have done in [23] and many others have done previously. We can also define a *fast-slow subsystem* of fast and slow variables together, and we can define separate fast and slow subsystems for the voltage compartment, since it includes slow $na_{i}$.

**Fig. 4:**
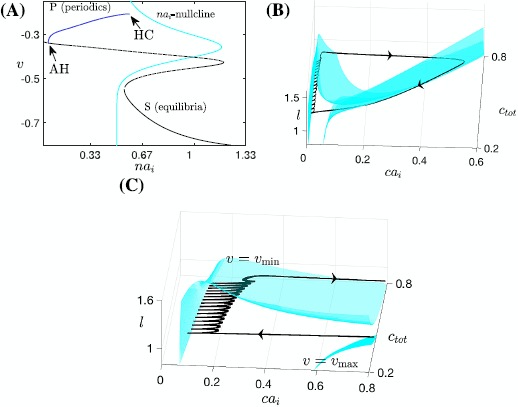
Basic structures of subsystems for the Jasinski model. Basic structures of subsystems for the Jasinski model (5a)–(5g). (**A**) Projection onto $({na_{i}}, v)$-space of the bifurcation diagram for the fast subsystem of the voltage compartment with $na_{i}$ as a bifurcation parameter, along with the $na_{i}$-nullcline shown in *cyan*. The *black curve* represents the critical manifold *S* of the fast subsystem (*solid* for stable fixed points, *dashed* for unstable), and the *blue curve* shows the maximum of *v* along the family of periodics *P*. (**B**) Nullsurfaces of $ca_{i}$ for the calcium compartment with *v* at its minimum (*upper surface*) and maximum (*lower surface*), in $(ca_{i}, c_{\mathrm{tot}}, l)$-space. The *black curve* denotes the SB solution trajectory of the nondimensionalized Jasinski model. The *right branches* of these two nullsurfaces lie close to each other. (**C**) A zoomed-in and enlarged view of (**B**)

The bifurcation diagram for the fast subsystem of the voltage compartment, comprising variables $(v, y, s)$ and decoupled from $ca_{i}$ by setting $\bar{g}_{\mathrm{Ca}}=\bar{g}_{\mathrm{CAN}}=0$, with the slow variable $na_{i}$ treated as a bifurcation parameter, is shown in Fig. 4A. It includes an S-shaped curve of equilibria (*S*) and a family of stable periodic orbits (*P*) that initiates in a supercritical Andronov–Hopf (AH) bifurcation and terminates in a homoclinic (HC) bifurcation involving the middle branch of *S* as $na_{i}$ is increased. Hence, in the absence of calcium dynamics, this subsystem is capable of generating a square-wave bursting solution, which terminates via the accumulation of $na_{i}$ and subsequent activation of the $\mathrm{Na}^{+}/\mathrm{K}^{+}$ pump. As part of our analysis of SB dynamics, we will in Sect. 3.1.1 consider what happens to this bursting, corresponding to the small bursts in the SB solution, once coupling from the calcium compartment to the voltage compartment is restored.

In the calcium compartment, the dynamics of $ca_{i}$ depends on the neuronal membrane potential *v*. We can represent this *v*-dependence by considering a family of $ca_{i}$ nullsurfaces, each defined for *v* fixed. While $ca_{i}$ is low, the projection of the trajectory to $(ca_{i}, c_{\mathrm{tot}}, l)$-space exhibits small oscillations during each regular burst (see Fig. 4C and the calcium trace in Fig. 3). These oscillations correspond to the projected trajectory trying to move back and forth between the left branches of two extreme nullsurfaces as *v* oscillates between its minimum and maximum during the spiking phase of each burst (Fig. 4B and C); the trajectory cannot make it all the way to the $v_{\max}$ surface because the dynamcs of $ca_{i}$ is slower than that of *v*. As for the right branches of these two extreme surfaces, Fig. 4B shows that they lie close together, which results because $I_{\mathrm{Ca}}$ depends only weakly on *v* for calcium large. As a result, if $ca_{i}$ is elevated, then the projected trajectory is constrained quite tightly between the two right branches of these nullsurfaces. We can observe that at the end of a cycle of the SB solution, the sigh-like burst is completed as the trajectory passes the curve of lower folds of the family of calcium nullsurfaces and jumps back to the left. What remains unclear about this loop is what bifurcation induces the jump-up of calcium, the understanding of which is crucial in illustrating the transition from regular bursts to the high-amplitude sigh-like burst. We consider this issue in Sect. 3.1.2, after first completing some additional analysis of the regular bursting phase with coupling from the calcium compartment to the voltage compartment restored.

#### Mechanisms Underlying Regular Bursting

**Fig. 5:**
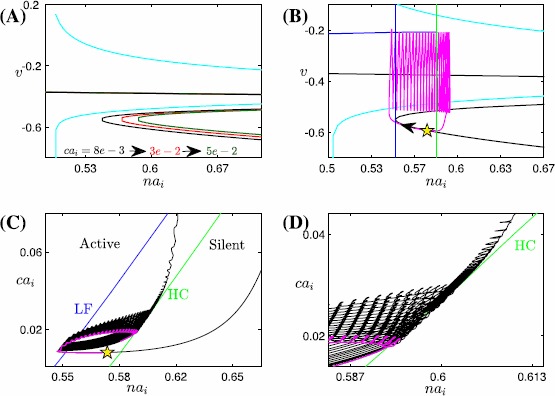
Bifurcation diagrams of the fast subsystem. Bifurcation diagrams of the fast subsystem with the slow variables $na_{i}$ and $ca_{i}$ taken as static parameters. The *yellow star* marks the start point of the SB solution. (**A**) The effect of $ca_{i}$ on the bifurcation diagram for the fast subsystem, projected into $(na_{i}, v)$-space, along with the $na_{i}$-nullcline (*cyan*). Increasing $ca_{i}$ from $8\mathrm{e}{-}3$ to $3\mathrm{e}{-}2$ to $5\mathrm{e}{-}2$ results in a shift of the bifurcation diagram to the *right* (*black to blue to green*). (**B**) Projection of the first small burst in the SB solution of (5a)–(5g) onto the bifurcation diagram (with $ca_{i}=8\mathrm{e}{-}3$) in $(na_{i}, v)$-space, along with the $na_{i}$-nullcline (*cyan*). The blue and green dashed lines indicate the $na_{i}$ values where the lower fold and homoclinic bifurcations occur, respectively. (**C**) The curve of saddle-node bifurcations corresponding to the lower fold of the bifurcation diagram (*blue*), homoclinic bifurcation curve (*green*) and part of the trajectory (*black*) generated by (5a)–(5g) in $(na_{i}, ca_{i})$-space. The HC curve splits the $(na_{i}, ca_{i})$-space into two regions labeled as ‘Active’ and ‘Silent’, respectively. The part of the trajectory corresponding to the first burst, as shown in (**B**), is magenta. (**D**) A zoomed-in and enlarged view of (**C**)

Setting $\bar{g}_{\mathrm{Ca}}=0.00065$ and $\bar{g}_{\mathrm{CAN}}=3$ as given in Table 3 restores the coupling from calcium to voltage and yields an SB solution. An example of the coupling effect on the voltage compartment can be seen in Fig. 5A: an increase of $ca_{i}$ shifts the $na_{i}$-dependent fast subsystem equilibria *S* to the right. In Fig. 5B, we project the first regular burst solution and the bifurcation diagram of the fast subsystem for $ca_{i}$ fixed at $8\mathrm{e}{-}3$, corresponding to its value at the beginning of this first small burst, onto $(na_{i}, v)$-space. Also shown is the green (resp. blue) dashed line representing the $na_{i}$ values at which the homoclinic (resp. lower fold of equilibria) bifurcation occurs. Starting from the yellow star, the trajectory moves on the slow timescale associated with $na_{i}$ along the stable lower branch of *S* until it reaches the lower fold. After that, the trajectory jumps up to the stable periodic orbit branch and then moves to the right, since the trajectory stays above the $na_{i}$-nullcline. Sometime after it crosses the homoclinic bifurcation at the $na_{i}$ value indicated by the green dashed line, the trajectory will jump down to the lower branch of equilibria, completing a small burst. This is essentially a square-wave burst, but notice that several more spikes occur after the green dashed line is passed. These spikes arise because during the first regular burst period, $ca_{i}$ progressively increases on a slow timescale; as a result, the bifurcation diagram also moves rightward on a slow timescale associated with the increase of $ca_{i}$ (Fig. 5A). Hence at the end of the burst, the homoclinic bifurcation actually occurs at some larger $na_{i}$ value to the right of the green dashed line in Fig. 5B, yielding several more spikes after the green dashed line. Such square-wave bursting solutions will repeat roughly until $ca_{i}$ starts to jump up to larger values as indicated in Figs. 3, 4.

Understanding the persistence of the regular bursts and the mechanism by which a transition to the sigh-like burst occurs requires us to consider the effect of $ca_{i}$ on the voltage compartment. To do so, we use $ca_{i}$ as the second bifurcation parameter and allow both $ca_{i}$ and $na_{i}$ to vary in order to find the two-parameter bifurcation curves of the fast subsystem $(v, y)$ in the $(na_{i}, ca_{i})$ parameter plane that unify the results in Fig. 5A, as illustrated in Fig. 5C. The blue (resp. green) curve in this plane is the curve of lower fold (LF) (resp. homoclinic (HC)) bifurcations, which initiates (resp. terminates) each burst, as noted previously. Since the increase of $ca_{i}$ moves the bifurcation diagram to the direction of increasing $na_{i}$, both the LF and the HC curves are positively sloped in $(na_{i}, ca_{i})$-space. Within the same projection, the trajectory evolves leftward from the yellow star and it starts oscillating as it passes the LF curve (see Fig. 5C). These oscillations terminate when the trajectory reaches the HC bifurcation curve, which completes the first regular burst. Similarly, a sequence of subsequent regular bursts occurs, with the local maximum of $na_{i}$ progressively increasing due to the rightward drift of LF as $ca_{i}$ accumulates. The fact that the trajectory in $(na_{i}, ca_{i})$-space crosses the LF and HC 15 times corresponds to the existence of 15 regular bursts between sighs (see Fig. 3). After these, bursting solutions give way to continuous spiking.

Based on the fast voltage compartment bifurcation structures in this section, we have seen that regular bursts occur as the slow variables $ca_{i}$ and $na_{i}$ traverse the phase space back and forth between the LF and HC curves. The reason why regular bursts switch to the sigh-like burst, however, has not been addressed. To figure this out, we notice that after multiple crossings of the HC curve and returns to quiescence in Fig. 5C, the trajectory projected to $(na_{i}, ca_{i})$ space starts oscillating near the HC curve, instead of going back again to the quiescent state (see Fig. 5D for an enlarged view of oscillations near the HC curve). Moreover, this transition happens before $ca_{i}$ jumps up. Hence, the switch from regular bursts to the long sigh-like burst in the full system seems to correspond to the transition from bursting to tonic spiking in the fast-slow subsystem $(v, y, na_{i}, ca_{i})$, rather than the jumping up of $ca_{i}$ in the calcium compartment as in the MB model [15]. We next consider the mechanism responsible for this transition.

#### Mechanisms Underlying the Transition from Regular Bursts to the Sigh-Like Burst

**Fig. 6:**
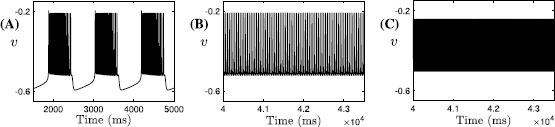
Effect of $c_{\mathrm{tot}}$ on the fast-slow subsystem. Effect of variations in $c_{\mathrm{tot}}$ on the trajectories of the fast-slow subsystem $(v, y, ca_{i}, na_{i})$ for $l=0.94$. In (**A**) $c_{\mathrm{tot}}=0.6$ (bursting), (**B**) $c_{\mathrm{tot}}=0.7$ (tonic spiking at low $ca_{i}$ and $na_{i}$: $ca_{i}\approx2.5\times10^{-2}$, $na_{i} \approx0.6$) and (**C**) $c_{\mathrm{tot}}=0.74$ (tonic spiking at high $ca_{i}$ and $na_{i}$: $ca_{i}\approx 0.57$ and $na_{i}\approx0.85$)

In the analysis up to this point, the superslow variables $c_{\mathrm{tot}}$ and *l* have not yet been considered. It is natural to expect that the superslow evolution of these two variables may contribute to the switch from bursting to tonic spiking. A numerical simulation of the fast-slow subsystem over a range of $c_{\mathrm{tot}}$ and *l* values (Fig. 6A–C) suggests that superslow variables do play an important role in inducing a transition from bursting to tonic spiking in the fast-slow subsystem. In Fig. 6A–C, representative time traces for bursting and tonic spiking solutions are shown for a fixed value of *l*. For small $c_{\mathrm{tot}}$, the fast-slow system is in a bursting state (Fig. 6A). When $c_{\mathrm{tot}}$ is increased, tonic spiking solutions arise (Fig. 6B). A further increase in $c_{\mathrm{tot}}$ accelerates the tonic spiking in *v* (Fig. 6C), and both $ca_{i}$ and $na_{i}$ oscillate at higher values than in (A) and (B).

**Fig. 7:**
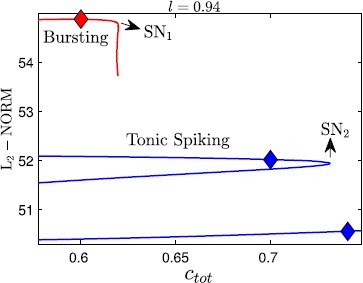
Bifurcation diagram of the fast-slow subsystem. The bifurcation diagram for the fast-slow subsystem with respect to the $c_{\mathrm{tot}}$ summarizes all possible behaviors as $c_{\mathrm{tot}}$ varies; *diamonds* mark $c_{\mathrm{tot}}$ values used in Fig. 6A–C

A graphical summary of the effect of $c_{\mathrm{tot}}$ variations on the trajectories is provided in Fig. 7, where the bifurcation structure of the fast-slow subsystem with respect to $c_{\mathrm{tot}}$ for $l=0.94$ is displayed. We plot the standard $L_{2}$ norm of the solution against $c_{\mathrm{tot}}$. The fast-slow system exhibits tristability between bursting (red solid) and two tonic spiking solutions (blue solid) for small $c_{\mathrm{tot}}$ values. As $c_{\mathrm{tot}}$ is increased, first the bursting branch becomes unstable at a saddle-node bifurcation and then one spiking branch does the same. At the start of an SB cycle, the trajectory is attracted by the stable bursting branch with $c_{\mathrm{tot}} \approx0.6$ (red diamond). Solution behavior switches to tonic spiking if $c_{\mathrm{tot}}$ is increased. Note that the lower $L_{2}$ norm corresponds to larger $ca_{i}$ and $na_{i}$ values; hence, as the trajectory gets attracted by the lower branch of spiking after the saddle-node bifurcation of the upper one, the tonic spiking solution occurs at larger $ca_{i}$, $na_{i}$ values.

Although Fig. 7 is suggestive, it remains to study more carefully the slow and superslow dynamics in order to understand the mechanisms underlying the switch from bursting to tonic spiking. To do so, we will average over the fast subsystem oscillations. For convenience, we refer to the two regions of $(na_{i}, ca_{i})$ space separated by the curve of HC bifurcations that terminates each regular burst as the silent and active regions, respectively (Fig. 5C).

During each interburst interval within the regular bursting epoch, the full model dynamically collapses to a lower-order system governed by the slow variables $ca_{i}$ and $na_{i}$ restricted to *S*, the manifold of equilibrium points of the fast subsystem. Each interburst interval occurs when the trajectory projected to $(na_{i}, ca_{i})$ space lies in the silent region. During the spiking phase of each regular burst, the solution trajectory is still largely determined by the slow variables $ca_{i}$ and $na_{i}$, but these variables are perturbed by the voltage spike and the $\mathrm{Ca}^{2+}$-influx associated with each action potential. This spiking phase corresponds to the active region of $(na_{i}, ca_{i})$ space. In this region we employ the method of averaging by numerically averaging the derivatives of the slow variables over one cycle of the action potential, while the superslow variables $c_{\mathrm{tot}}$ and *l* are treated as static parameters. By doing so, we reduce the fast-slow subsystem to two equations for just the slow variables. For $g_{1}$ and $g_{2}$ defined as the right-hand sides of (5d) and (5f), respectively, the reduced system can be written as

6a $$\begin{aligned} R_{ca_{i}}\langle\dot{ca_{i}}\rangle =&\frac{1}{T(ca_{i}, na_{i})} \int_{0}^{T(ca_{i}, na_{i})}g_{1}\bigl(v(ca_{i}, na_{i}; t), ca_{i}, c_{\mathrm{tot}}, l\bigr)\, dt, \end{aligned}$$

6b $$\begin{aligned} R_{na_{i}}\langle \dot{na_{i}}\rangle =&\frac{1}{T(ca_{i}, na_{i})} \int_{0}^{T(ca_{i}, na_{i})}g_{2}\bigl(v(ca_{i}, na_{i}; t), na_{i}, ca_{i}\bigr)\, dt. \end{aligned}$$

We refer to the reduced problem (6a)–(6b) as the *averaged slow system*. The nullclines of the averaged slow system are curves of $(na_{i}, ca_{i})$ values along which there exist periodic solutions (with period $T(ca_{i}, na_{i})$) of the fast-slow subsystem that satisfy the additional constraint of either $\langle\dot{ca_{i}}\rangle=0$ or $\langle\dot {na_{i}}\rangle=0$. In future discussions of the dynamics of the averaged slow system, we will refer to the $ca_{i}$ and $na_{i}$ average nullclines as $ca_{\mathrm{av}}$ and $na_{\mathrm{av}}$, respectively. Each intersection of $ca_{\mathrm{av}}$ and $na_{\mathrm{av}}$ is a fixed point of system (6a)–(6b) representing a tonic spiking solution of the fast-slow subsystem, which we will refer to as $\mathrm{FP}_{\mathrm{av}i}$ for some index *i*.

**Fig. 8:**
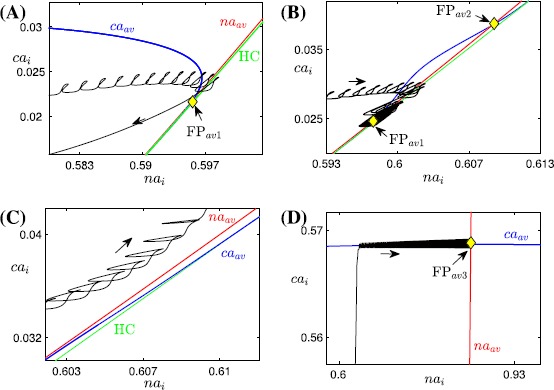
Averaged phase planes. Averaged phase planes, corresponding to system (6a)–(6b), with superimposed trajectories of the fast-slow system, for $l=0.94$ and three different values of $c_{\mathrm{tot}}$ as in Fig. 6. Throughout this figure, the curve of HC bifurcations is *green*, the nullclines $ca_{\mathrm{av}}$, $na_{\mathrm{av}}$ are *blue* and *red*, respectively, and the *yellow symbols* mark the fixed points of (6a)–(6b). The oscillatory trajectories (*black*) from Fig. 6A–C are projected to $(na_{i}, ca_{i})$-space in (**A**), (**B**), and the *lower two panels* in this figure, respectively. For (**A**) $c_{\mathrm{tot}}=0.6$ and (**B**) $c_{\mathrm{tot}}=0.7$, there are three fixed points of (6a)–(6b) above HC where the average nullclines intersect, namely, $\mathrm{FP}_{\mathrm{av}1}$, $\mathrm{FP}_{\mathrm{av}3}$, which are stable, and $\mathrm{FP}_{\mathrm{av}2}$, which is unstable. Not all fixed points are visible since some of them lie at larger $( na_{i}, ca_{i})$ values than those shown. (**C**) and (**D**) Enlarged views of the phase plane for $c_{\mathrm{tot}}=0.74$ showing that both $\mathrm{FP}_{\mathrm{av}1}$ and $\mathrm{FP}_{\mathrm{av}2}$ vanish and the only average fixed point left ($\mathrm{FP}_{\mathrm{av}3}$, with high $na_{i}$ and $ca_{i}$) is stable. Specifically, panel (**C**) (resp. (**D**)) shows the enlarged *lower left* (resp. *upper right*) *region* of the phase plane; note differences in values on the axes

Figure 8 illustrates phase planes of the average slow system (6a)–(6b) for $l=0.94$ and $c_{\mathrm{tot}}=0.6, 0.7, 0.74$ as in Fig. 6. In each panel of Fig. 8, the green curve represents the HC bifurcation of the fast subsystem that forms the boundary of the oscillation region. Above HC, where the fast subsystem oscillates (Fig. 5C), the averaged nullclines $ca_{\mathrm{av}}$ (blue curve) and $na_{\mathrm{av}}$ (green curve) are shown. As noted before, fixed points of (6a)–(6b), $\mathrm{FP}_{\mathrm{av}i}$ (yellow diamonds), are given by the intersections of these nullclines, and one can usually determine the stability of the fixed points by considering the nullcline configuration.

In Fig. 8A with $c_{\mathrm{tot}}=0.6$, the two average nullclines intersect at a stable fixed point $\mathrm{FP}_{\mathrm{av}1}$ (yellow diamond), which corresponds to the upper spiking branch in Fig. 6D. Despite the existence of this stable fixed point (corresponding to stable tonic spiking), the fast-slow subsystem exhibits bursting since our chosen initial values lie in the basin of attraction of the bursting branch. Correspondingly, in Fig. 8A, the projected trajectory moves clockwise, exhibiting small loops corresponding to spikes within a regular burst, until it crosses HC, at which point the regular burst terminates and the loops are lost while the trajectory transits along a stable branch of the equilibrium curve *S* (not shown here).

At $c_{\mathrm{tot}}=0.7$, the stable bursting branch has been lost (Fig. 7) and hence the trajectory is now attracted by the stable fixed point $\mathrm{FP}_{\mathrm{av}1}$ (Fig. 8B, yellow diamond). There are also a saddle equilibrium $\mathrm{FP}_{\mathrm{av}2}$, visible in the figure, and a third fixed point of (6a)–(6b) that lies at larger $ca_{i}$ and $na_{i}$ values, not shown here. As a result, the fast-slow subsystem converges to the lower stable fixed point $\mathrm{FP}_{\mathrm{av}1}$ and exhibits tonic spiking.

As $c_{\mathrm{tot}}$ increases further to 0.74, the lower two fixed points $\mathrm{FP}_{\mathrm{av}1}$ and $\mathrm{FP}_{\mathrm{av}2}$ collide and annihilate through a saddle-node bifurcation ($\mathrm{SN}_{2}$ in Fig. 7; note their absence in Fig. 8C) and only the upper stable fixed point $\mathrm{FP}_{\mathrm{av}3}$ remains (Fig. 8D, yellow diamond), corresponding to the lower spiking branch in Fig. 7. Therefore, the trajectory jumps up to large $ca_{i}$ values on the slow timescale until it reaches a small neighborhood of the average $ca_{i}$ nullcline (blue curve in Fig. 8D). Once there, the trajectory approaches the fixed point $\mathrm{FP}_{\mathrm{av}3}$ since $\langle \dot{na_{i}}\rangle$ remains positive. As the trajectory converges toward $\mathrm{FP}_{\mathrm{av}3}$, tonic spiking dynamics at large $ca_{i}$ and $na_{i}$ values results.

**Fig. 9:**
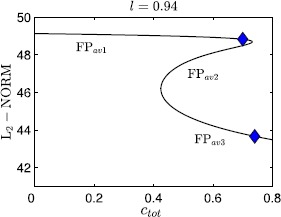
Bifurcation diagram of the average slow system. Bifurcation diagram of the average slow system (6a)–(6b) with respect to $c_{\mathrm{tot}}$ with *l* fixed at 0.94. The $c_{\mathrm{tot}}$ values for the *blue diamond marker* points are $c_{\mathrm{tot}}=0.7$ (*upper*; as in Fig. 8B) and 0.74 (*lower*; as in Fig. 8C, D)

Using slow averaged dynamics, we have elucidated how the transition from bursting to tonic spiking occurs and why $ca_{i}$ jumps to larger values as $c_{\mathrm{tot}}$ increases for *l* fixed. Similarly, we summarize the effects of $c_{\mathrm{tot}}$ on the average slow system by using bifurcation analysis as shown in Fig. 9. Again, the upper (resp. lower) branch of the bifurcation diagram in $L_{2}$ norm corresponds to lower (resp. upper) values of $ca_{i}$ as well as $na_{i}$ and hence solutions on this branch denote $\mathrm{FP}_{\mathrm{av}1}$ (resp. $\mathrm{FP}_{\mathrm{av}3}$). The middle branch represents the unstable saddle $\mathrm{FP}_{\mathrm{av}2}$ (see Fig. 8B). Notice that Fig. 9 looks qualitatively the same as the tonic spiking curves shown in blue in Fig. 7, hence either can be used to illustrate the influence of $c_{\mathrm{tot}}$ on the oscillatory trajectories for a fixed value of *l*. As $c_{\mathrm{tot}}$ is increased, calcium jumps up at a saddle-node (SN) seen both in the tonic spiking branch in Fig. 7 and in the curve of average system fixed points in Fig. 9. On the other hand, the onset of spiking happens at a SN of the bursting branch (Fig. 7, red). Next we extend this bifurcation analysis and examine the dependence of the solution patterns of the fast-slow subsystem on both superslow variables $c_{\mathrm{tot}}$ and *l*. To do this, we compute two-parameter bifurcation diagrams in $(c_{\mathrm{tot}}, l)$-space.

**Fig. 10:**
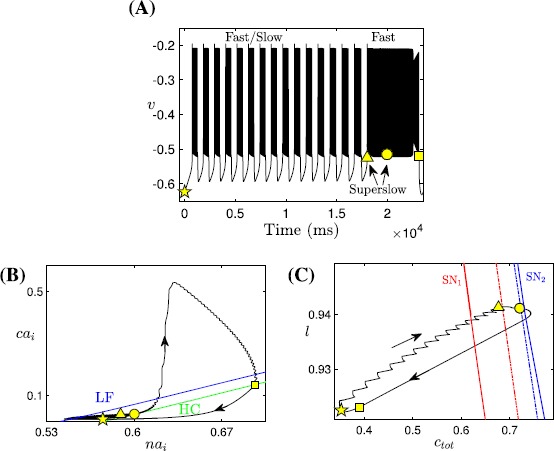
Mechnisms underlying sigh-like bursting solutions. Simulation of the SB solution generated by (5a)–(5g), together with the bifurcation diagrams. (**A**) Temporal evolution of *v*. *Yellow symbols* mark key points along the solution (*star*: start of the SB solution as in Fig. 5; *triangle*: start of the sigh-like burst; *circle*: start of jumping up of $ca_{i}$; *square*: termination of the sigh-like burst). (**B**) Two-parameter bifurcation diagrams showing LF (*blue*) and HC (*green*) curves together with the trajectory from panel (**A**) in $(na_{i}, ca_{i})$-space, the enlarged view of the lower left part of which is given by Fig. 5C. (**C**) The curves of saddle-node bifurcations corresponding to the upper folds of the bursting branch and the spiking branch in Fig. 7, denoted as $\mathrm{SN}_{1}$ and $\mathrm{SN}_{2}$, respectively. We use $\mathrm{SN}_{1}$ (*solid red*) to approximate the bursting/spiking boundary for the fast-slow subsystem (*dashed red*) and use $\mathrm{SN}_{2}$ (*solid blue*) to approximate the onset of jumping up of $ca_{i}$ (*dashed blue*), respectively

In Fig. 10C, the fast-slow subsystem spiking/bursting boundary (solid red, $\mathrm{SN}_{1}$) was calculated by following in the two superslow variables $(c_{\mathrm{tot}}, l)$ the SN point where the bursting branch loses stability (Fig. 7, red curve). Also shown is the boundary (solid blue, $\mathrm{SN}_{2}$) demarcating where $ca_{i}$ jumps up, computed by following the upper fold point of the tonic spiking branch in Fig. 7. We can also use direct simulation of the fast-slow subsystem (e.g., fixing $c_{\mathrm{tot}}$, varying *l* systematically, and then repeating for a different $c_{\mathrm{tot}}$), to estimate the bursting/spiking boundary curve and the onset of the jump-up in calcium (shown in dashed red and blue). The dashed curves approach the solid ones as we exaggerate the timescale separation between the fast-slow subsystem and the superslow variables (data not shown).

Combining these results with the fast subsystem bifurcation analysis as illustrated in Fig. 5C, and reproduced in Fig. 10B, we are now able to fully understand the SB solution shown in Fig. 10A. Starting from the yellow star at low $c_{\mathrm{tot}}$ and *l* (Fig. 10), a sequence of small bursts is produced as the trajectory in $(ca_{i}, na_{i})$-space oscillates between the LF and HC curves multiple times (Fig. 10B). A key step toward termination of this process occurs when the increases of $c_{\mathrm{tot}}$ and *l* push the trajectory in $(c_{\mathrm{tot}},l)$-space across the $\mathrm{SN}_{1}$, such that the fast-slow subsystem enters the tonic spiking regime. In fact, several more small bursts actually occur after the crossing of $\mathrm{SN}_{1}$ and are followed by the start of tonic spiking at the triangle. In the singular limit, however, this additional bursting will be lost and the tonic spiking occurs when the trajectory reaches $\mathrm{SN}_{1}$. Within the same projection, the trajectory evolves rightward from the triangle and eventually passes the $\mathrm{SN}_{2}$ curve at some point close to the yellow circle, which initiates the jump-up of $ca_{i}$. The increase in $ca_{i}$ as well as in $na_{i}$ from the yellow circle, which occur on the slow timescale, can be seen in the projection shown in Fig. 10B. Note that this jump corresponds in the projection into $(ca_{i}, c_{\mathrm{tot}}, l)$-space to the convergence of the trajectory to the right branches of the $ca_{i}$-nullsurface family (Fig. 4B). For $ca_{i}$ large, the trajectory in $(ca_{i}, c_{\mathrm{tot}}, l)$-space lies above the *l*-nullsurface (not shown here); consequently, *l* next decreases (corresponding also to the decrease in *l* in Fig. 10C), leading to the reduction of $ca_{i}$. Therefore, the trajectory in $(na_{i}, ca_{i})$-space falls back from the peak in $ca_{i}$ direction and moves towards HC (Fig. 10B). Once it crosses the HC bifurcation curve at the yellow square, the long burst ends and the trajectory enters the silent phase. As the solution finally returns to its starting point (yellow star), one cycle of the SB solution is completed.

##### Remark 1

Some time after $ca_{i}$ jumps up at the yellow circle, the amplitude of the *v* spikes exhibits a sudden decrease followed by a gradual increase (Fig. 10A). This behavior arises because periodic orbits of the voltage compartment in the Jasinski model initiate in an AH bifurcation with zero amplitude, while orbit amplitudes increase closer to the HC bifurcation (Fig. 4A). Therefore, the sudden decrease of the amplitude of *v* spikes results from the fact that the jump-up of $ca_{i}$ pushes the trajectory away from the HC curve and closer to the AH. The subsequent decrease in $ca_{i}$ yields a return toward the HC, leading to the gradual increase in the amplitude. This mechanism is the same as observed in MB solutions in [15].

### Identifying Timescales

MB solutions, studied previously [15], involve gradual transitions between two different types of bursts, like the SB solutions that we are now considering but with different underlying biological mechanisms. Surprisingly, we obtained the non-intuitive result that the existence of robust MB solutions does not require a third timescale. Thus, a natural question is: how many timescales are fundamentally important for generating SB solutions? To address this question, we adopt the approach used in [23] of transforming our original system into certain two-timescale systems by adjusting system parameters (see Table 5 in Appendix 1). Then we consider whether SB solutions can persist under these adjustments.

The Jasinski model has 7 fast, 2 slow, and 2 superslow (7F, 2S, 2SS) variables. In theory, the timescale separation between some of these groups might not be necessary to generate SB dynamics. Thus, we will consider what happens if we group together the fast and slow variables to form a (9F, 2SS) system and what happens if we group together the slow and superslow variables to form a (7F, 4SS) system. To do so, we first choose $A=1$, so that *l* evolves on a comparable timescale to the other superslow variable, $c_{\mathrm{tot}}$. Since parameters that control the timescale for $ca_{i}$ will also affect the timescale for $c_{\mathrm{tot}}$, we introduce a new parameter, *β*, with default value 1, as a scaling factor specifically for the right-hand side of the $ca_{i}$ Eq. (1d). To change the timescale for $na_{i}$, we vary $\alpha_{\mathrm{Na}}$. We will form our (9F, 2SS) system by increasing both *β* and $\alpha_{\mathrm{Na}}$ by a factor of 100, and we will form our (7F, 4SS) system by reducing both *β* and $\alpha _{\mathrm{Na}}$ by a factor of 10.

**Fig. 11:**
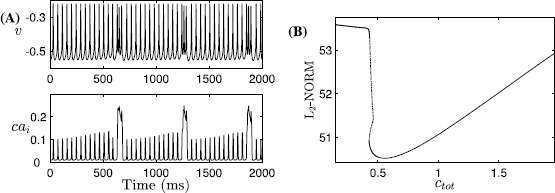
Simulations of the Jasinski model with two-time-scale reduction. Rescaled version of (5a)–(5g): (9F, 2SS) case, with $\beta =100$, $\alpha_{\mathrm{Na}}=5\times10^{-3}$. (**A**) Time series of *v* and $ca_{i}$. (**B**) The bifurcation diagram for the 9-dimensional layer problem of the (9F, 2SS) system, with bifurcation parameter $c_{\mathrm{tot}}$ and $l=0.94$ fixed. *Solid curves* denote stable tonic spiking solutions while the *dashed curve* denotes unstable solutions. At least one such solution is present for all $c_{\mathrm{tot}}$; in fact, the stable branches overlap over a small interval in $c_{\mathrm{tot}}$, yielding bistability

With its original scaling, system (5a)–(5g) generates a SB solution, as shown in Fig. 10A. Increasing *A* to 1 does not change this solution qualitatively, except that the number of small bursts decreases. That is, as *l* becomes faster, the trajectory projected into $(c_{\mathrm{tot}}, l)$-space will reach the spiking/bursting boundary curve ($\mathrm{SN}_{1}$) earlier and hence small bursts give way to a long burst earlier (Fig. 10C). We next compare this SB solution to solutions from the (9F, 2SS) version of the system (5a)–(5g) described above (compare Fig. 10A with Fig. 11A). The fast/slow decomposition method (between $(v, y)$ and $(na_{i}, ca_{i})$ for understanding the mechanisms underlying the regular bursts within the SB solution no longer applies to the (9F, 2SS) case, because $ca_{i}$ and $na_{i}$ now evolve on the fast timescale. In contrast to the original (7F, 2S, 2SS) case where the (7F, 2S)-subsystem generates bursting solutions for relatively small $c_{\mathrm{tot}}$ and *l* values (see Figs. 6 and 7), the new 9-dimensional fast subsystem for the (9F, 2SS) case exhibits tonic spiking for all $c_{\mathrm{tot}}$ and *l* values within a complete bursting cycle. The effect of $c_{\mathrm{tot}}$ on the fast subsystem trajectories can be determined from Fig. 11B, where the bifurcation diagram of the fast subsystem (9F) with respect to $c_{\mathrm{tot}}$ for $l=0.94$ is displayed. Two branches of stable solutions are present, both corresponding to tonic spiking, and one or more stable tonic spiking solutions exist for all $c_{\mathrm{tot}}$ values, while the bursting branch that was found in the original system no longer exists in this case. The stable branches persist in two-parameter $(c_{\mathrm{tot}}, l)$-space for all relevant *l* (data not shown); therefore, as $c_{\mathrm{tot}}, l$ evolve on the superslow timescale, the trajectory remains on these spiking branches and spiking persists for all time, and the system cannot sustain a SB solution.

**Fig. 12:**
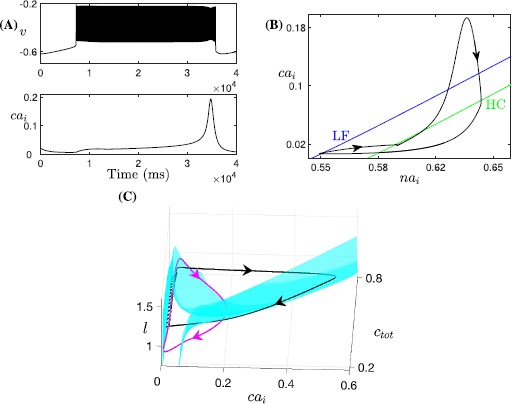
Simulations of the Jasinski model with two-time-scale reduction. Rescaled version of (5a)–(5g): (7F, 4SS) case, with $\beta =0.1$, $\alpha_{\mathrm{Na}}=5\times10^{-6}$. (**A**) Time series of *v* and $ca_{i}$. (**B**) The projection of the trajectory from (**A**) onto a two-parameter bifurcation diagram for the 7-dimensional layer problem of the (7F, 4SS) system. (**C**) Projections to $(ca_{i}, l, c_{\mathrm{tot}})$-space of the $ca_{i}$-nullsurfaces and (7F, 2S, 2SS) trajectory (*black*) as illustrated in Fig. 4B and C, together with the (7F, 4SS) trajectory (*magenta*) from (**A**), (**B**)

Under the alternative rescaling to a (7F, 4SS) system, one cycle of the bursting solution is as shown in Fig. 12A. Only long sigh-like bursts now occur, without regular bursts. Note that the (7F, 2S, 2SS) and (7F, 4SS) systems have the same fast subsystem, with the same LF and HC curves in $(na_{i}, ca_{i})$-space (Figs. 10, 12B). Within the projection into the $(na_{i}, ca_{i})$-space, a burst of activity begins as the trajectory evolves clockwise in the direction of increasing $ca_{i}$ and $na_{i}$ from the LF curve in the lower left part of the figure. Eventually, $c_{\mathrm{tot}}$, *l* change enough to cause a rise in the target value of $ca_{i}$; the details differ from the (7F, 2S, 2SS) case because the slow averaged dynamics and fast-slow subsystem are no longer relevant, but the outcome is similar. With the (7F, 4SS) rescaling, $ca_{i}$, $na_{i}$ evolve on the same superslow time scale as $c_{\mathrm{tot}}$, *l*. Hence, the drift of the trajectory before this transition is too slow for the solution to reach the curve of HC bifurcations and fall silent. As a result, the single burst continues all the way up until the transition; that is, regular bursting never occurs. While the burst continues, all four superslow variables increase until the trajectory projected to $(ca_{i}, l, c_{\mathrm{tot}})$-space goes above the *l*-nullsurface (not shown here). After that, *l* starts decreasing, which eventually leads to the decrease in $ca_{i}$ (Fig. 12B,C). Again similarly as before, this reduction in $ca_{i}$ is fundamental in terminating the burst as it brings the trajectory across the curve of HC bifurcations (Fig. 12B). Afterwards, the solution enters the silent phase and goes back to the starting point, completing one period consisting simply of one single long burst.

In summary, neither of these two-timescale systems, despite the fact that they are the ones with the most similarity to the full three-timescale system, captures the full features of the SB solution shown in Fig. 10A. It is critical that $ca_{i}$, $na_{i}$ are distinctly slower than the fast voltage and other variables and faster than $c_{\mathrm{tot}}$, *l* for regular bursts between sighs to occur. We thus conclude that presence of three timescales is necessary for the emergence of the type of the SB solution we have studied, which differs from what was obtained in the pre-BötC MB model [15].

## Sigh-Like Spiking in a Single Pre-BötC Neuron

In Sect. 3, we have used the fast-slow decomposition method, bifurcation analysis and the averaging method to understand the mechanisms underlying the SB solution pattern, from which we discover that the short, eupneic bursts are of the well-known square-wave or fold-homoclinic type [16]. A square-wave burster is a kind of relaxation oscillator except that its active state comprises a fast oscillation rather than a quasi-steady plateau. For a square-wave burster, we can convert the upper active or spiking state to a quasi-steady state by removing specifically the fast-spiking components from the model. Following this logic, since the SS solution is composed of a large spike emerging periodically from a pattern of ongoing regular spikes, we may think of the SS solution as a simplification of the SB solution in which fast-spiking components are removed from all active states.

In fact, the discussion in Sect. 2.2 suggests that instead of working with the full SS pattern containing eupneic spikes (Fig. 2A), we can focus entirely on the activity of the sigh compartment (Fig. 2B). Thus, we simply need to analyze what happens during the prolonged silent phase and how the transition to the large, sigh-like spike occurs, paralleling the analysis in Sect. 3. We will first analyze what timescales are present in the model in the regime that supports the SS solutions of Fig. 2B and how they should be grouped to apply geometric singular perturbation theory. After applying these methods, we will investigate how many timescales are truly required in (3a)–(3e) in order to obtain these SS solutions.

We begin with nondimensionalization, defining new dimensionless variables $(v, ca_{i}, c_{\mathrm{tot}}, \tau)$ and voltage, calcium and time scales $Q_{v}$, $Q_{c}$ and $Q_{t}$:

$$V=Q_{v}\cdot v, \qquad \mathrm{Ca}_{\mathrm{i}}=Q_{c} \cdot ca_{i},\qquad \mathrm {Ca}_{\mathrm{tot}}=Q_{c}\cdot c_{\mathrm{tot}},\qquad t=Q_{t}\cdot t_{s}; $$

note that *h* and *l* are already dimensionless in (3a)–(3e). Details of the nondimensionalization procedure, including the determination of appropriate values for $Q_{v}$, $Q_{c}$ and $Q_{t}$, are given in Appendix 2. From this process, we obtain a dimensionless system of the form

7a $$\begin{aligned} \varepsilon\frac{dv}{dt_{s}} =&\hat{f}_{1}(v, h, ca_{i}), \end{aligned}$$

7b $$\begin{aligned} \frac{dh}{dt_{s}} =&\hat{h}_{1}(v, h), \end{aligned}$$

7c $$\begin{aligned} \frac{d c_{\mathrm{tot}}}{dt_{s}} =&\delta\hat{g}_{1}(v)+d\delta\hat {g}_{2}(ca_{i}):= \hat{g}(v,ca_{i}), \end{aligned}$$

7d $$\begin{aligned} \varepsilon\frac{dca_{i}}{dt_{s}} =&\hat{f}_{2}(v,ca_{i}, c_{\mathrm{tot}},l), \end{aligned}$$

7e $$\begin{aligned} \frac{dl}{dt_{s}} =&\hat{g}_{3}(ca_{i}, l) , \end{aligned}$$

where $\hat{f}_{1}$, $\hat{h}_{1}$, $\hat{g}_{1}$, $\hat{g}_{2}$, $\hat {f}_{2}$, $\hat{g}_{3}$ are $O(1)$ functions and *ε*, *δ* are small parameters. *d* has size $O(1)$ for *c* small (during the silent phase) and approximately $1/\delta$ for *c* large (during the active phase). From this nondimensionalization result, we conclude that *v* and $ca_{i}$ evolve on a fast timescale, and *h* and *l* evolve on a slow timescale. $c_{\mathrm{tot}}$, however, has different evolution rates at different times: it evolves on a slow timescale for *c* large because $\hat{g}\approx\delta\hat{g}_{1}+\hat{g}_{2}$ is an $O(1)$ function, and it evolves on a superslow timescale for *c* small since $\hat {g}\approx\delta(\hat{g}_{1}+\hat{g}_{2})$ is approximately an $O(\delta)$ function.

**Fig. 13:**
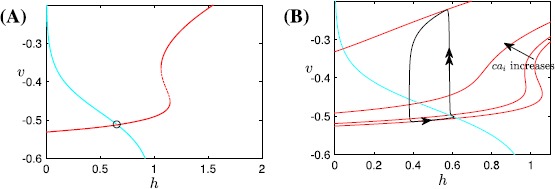
Impact of $ca_{i}$ on $(v, h)$ system. Impact of $ca_{i}$ on the nullclines and fixed points of the $(v, h)$ system. (**A**) *v*-nullcline (*red*) and *h*-nullcline (*cyan*) of the layer system (8a)–(8b) intersect at a single stable fixed point denoted by the *circle*. (**B**) Increasing $ca_{i}$ moves the *v*-nullcline to the upper left and eventually induces a cusp bifurcation but a unique fixed point remains for all $ca_{i}$. A solution trajectory of (7a)–(7e) projected into $(v,h)$-space (*black*) stays near the stable fixed point location for a transient period and then jumps away

The SS system features bidirectional coupling between *v* and $ca_{i}$, which is similar to that in the Jasinski model; therefore, we may also be able to explain the mechanisms underlying SS dynamics from the perspective of how the $(v, h)$ system is driven by the $(ca_{i}, c_{\mathrm{tot}}, l)$ system in analogy to our approach for SB solutions in Sect. 3. In the absence of coupling, the $(v,h)$ system has a unique stable equilibrium and no stable oscillatory solution (see Fig. 13A). When the coupling is restored by making $g_{\mathrm{Ca}}$ and $g_{\mathrm{CAN}}$ nonzero as given in Table 4, the increase of $ca_{i}$ moves the *v*-nullcline to the upper left (Fig. 13B, red). Meanwhile, the two folds of the nullcline meet and disappear as $ca_{i}$ is made larger than roughly 0.0376. As a result, the unique fixed point of the $(v, h)$ system remains stable for $ca_{i}\rightarrow\infty$. That is, there is no value at which $ca_{i}$ can be fixed to yield stable oscillations in $(v, h)$. However, although the fixed point in $(v, h)$ remains stable for all $ca_{i}$, the SS trajectory projected into $(v, h)$-space jumps away from the family of fixed points, to larger *v*, after staying nearby for a finite time. Therefore, we see that the onset of *v*-spike does not result from the variation of $ca_{i}$ pushing the $(v, h)$ system through any bifurcation at which oscillations are born. To understand how the increase of $ca_{i}$ triggers the fast jump in *v*, we notice that $ca_{i}$ can be considered to be as fast as *v*, rather than a slow variable, when it is not near its nullsurface and thus slaved to the slower variables *l* and $c_{\mathrm{tot}}$. This analysis confirms that since $ca_{i}$ jumps up on a fast timescale, the bifurcation diagram with $ca_{i}$ treated as a static parameter as shown in Fig. 13 no longer plays a role. Hence, a different subsystem classification is needed to explain the SS dynamics.

We use GSPT to define an array of subsystems for system (7a)–(7e), following [23]. Introducing a fast time $t_{f}=t_{s}/\varepsilon$ and letting $\varepsilon \rightarrow0$, we can derive a 2-dimensional fast layer problem that describes the dynamics of the fast variables *v* and $ca_{i}$, for fixed values of the other variables:

8a $$\begin{aligned} \frac{dv}{dt_{f}} =&\hat{f}_{1}(v,h,ca_{i}), \end{aligned}$$

8b $$\begin{aligned} \frac{d ca_{i}}{dt_{f}} =& \hat{f}_{2}(v,ca_{i},c_{\mathrm{tot}},l). \end{aligned}$$

We define the critical manifold $\mathscr {M}_{s}$ to be the manifold of equilibrium points of the fast layer problem, i.e.,

$$\mathscr {M}_{s}:=\bigl\{ (v, h, ca_{i}, c_{\mathrm{tot}},l): \hat{f}_{1}= \hat{f}_{2}=0\bigr\} . $$

Obtaining slow reduced problems is trickier here since $c_{\mathrm{tot}}$ has different scalings at different times. During the silent phase where $ca_{i}$ is relatively small, taking singular limits $\varepsilon, \delta\rightarrow0$ in (7a)–(7e) yields a system that describes the dynamics of the slow variables *h* and *l* for fixed values of $c_{\mathrm{tot}}$, with all variables restricted to the surface of $\mathscr {M}_{s}$,

9a $$\begin{aligned} \frac{dh}{dt_{s}} =&\hat{h}_{1}(v,h), \end{aligned}$$

9b $$\begin{aligned} \frac{d l}{dt_{s}} =& \hat{g}_{3}(ca_{i},l), \end{aligned}$$

subject to the constraint $\hat{f}_{1}=\hat{f}_{2}=0$. Following the terminology used in [23], we call this system the *slow reduced layer problem* and we define the superslow manifold $\mathscr {M}_{ss}$ to be the manifold of its equilibrium points, i.e.,

$$\mathscr {M}_{ss}:=\bigl\{ (v, h, ca_{i}, c_{\mathrm{tot}},l): \hat{f}_{1}=\hat{f}_{2}=\hat{h}_{1}=\hat {g}_{3}=0\bigr\} \subset \mathscr {M}_{s}. $$

To describe the dynamics of $c_{\mathrm{tot}}$ restricted to $\mathscr {M}_{ss}$, we define a superslow time $t_{ss}=\delta t_{s}$ and rewrite (7a)–(7e) as a rescaled system with respect to $t_{ss}$. Taking the singular limits $\varepsilon ,\delta\rightarrow0$ in the rescaled system yields the *superslow reduced problem*:

10 $$ \frac{dc_{\mathrm{tot}}}{dt_{ss}} = \hat{g}_{1}(v)+d \hat{g}_{2}(ca_{i}). $$

On the other hand, during the active phase when $ca_{i}$ is relatively large, we have $\hat{g}(v, ca_{i})=O(1)$. In other words, $c_{\mathrm{tot}}$ evolves on a slow timescale. In this case, taking the limits $\varepsilon, \delta\rightarrow0$ in (7a)–(7e) gives a system that describes the dynamics of all three slow variables *h*, $c_{\mathrm{tot}}$ and *l*,

11a $$\begin{aligned} \frac{dh}{dt_{s}} =&\hat{h}_{1}(v,h), \end{aligned}$$

11b $$\begin{aligned} \frac{dc_{\mathrm{tot}}}{dt_{s}} =& \hat{g}_{2}(ca_{i}), \end{aligned}$$

11c $$\begin{aligned} \frac{d l}{dt_{s}} =& \hat{g}_{3}(ca_{i},l), \end{aligned}$$

subject to the constraint $\hat{f}_{1}=\hat{f}_{2}=0$. We call system (11a)–(11c) the *slow reduced problem*.

Finally, we can also define a *fast-slow subsystem* of fast and slow variables together, as we did in Sect. 3.1. With these timescale groupings and subsystems defined, we can proceed to analyze the mechanisms underlying the SS solutions.

### Analysis of the SS Solutions

**Fig. 14:**
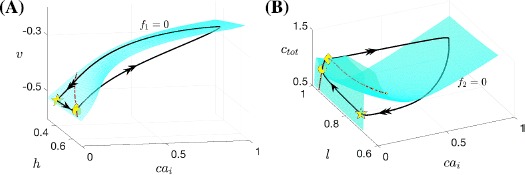
Mechanisms underlying the sigh-like spiking solution. A sigh-like spiking solution of (7a)–(7e) (*black curve*), projected to (**A**) $(v, h, ca_{i})$-space and (**B**) $(c, c_{\mathrm{tot}}, l)$-space. Also shown are projections of $\mathscr {M}_{s}$ (*colored surface*) and $\mathscr {M}_{ss}$ (*red curves*, *solid* where attracting, *dashed* otherwise). The *yellow star* and *circle* indicate points where the trajectory approximately reaches $\mathscr {M}_{s}$ and $\mathscr {M}_{ss}$, respectively. The *yellow diamond* in $\mathscr {M}_{ss}$ represents the subcritical AH bifurcation of the fast-slow subsystem $(v, h, ca_{i},l)$

The GSPT approach starts with a bifurcation analysis of the layer problem (8a)–(8b), which requires a visualization of the set of equilibria of the layer problem, $\mathscr {M}_{s}$, given by $\hat{f}_{1}(v, h, ca_{i})=\hat{f}_{2}(v, ca_{i}, c_{\mathrm{tot}}, l)=0$. Since the phase space for the full nondimensionalized system (7a)–(7e) is 5-dimensional, $\mathscr {M}_{s}$ is a 3-dimensional manifold. Since $\hat{f}_{2}$ does not depend on *h*, the projection of $\mathscr {M}_{s}$ onto $(v, h, ca_{i})$-space is simply given by $\hat{f}_{1}(v, h, ca_{i})=0$; that is, for all relevant $(v, h, ca_{i})$, we can solve $\hat{f}_{2}=0$ for an appropriate choice of $(c_{\mathrm{tot}}, l)$. We can solve $\hat{f}_{1}=0$ for *h* as a function of *v* and $ca_{i}$ and can therefore represent $\mathscr {M}_{s}$ projected onto $(v, h, ca_{i})$-space as $h=F_{1}(v, ca_{i})$ for a function $F_{1}$ (Fig. 14A). For each fixed $ca_{i}$ value, $\hat{f}_{1}=0$ is represented by a single curve, as shown in Fig. 13B.

#### Remark 2

For the purpose of nondimensionalization in Appendix 2, we require that $c_{\mathrm{tot}} \in[0,1]$. However, technically, the value of $c_{\mathrm{tot}}$ needed to make $\hat{f}_{2}(v, ca_{i}, c_{\mathrm{tot}},l)=0$ for some relevant $(v,h, ca_{i})$ can be greater than 1, but only as large as 1.5. Since this number is just marginally larger than 1, adjusting our nondimensionalization in Appendix 2 accordingly would not change the scales of any variables, and hence this is just a technicality that can be ignored for practical purposes.

Further insight into the dynamics of calcium comes from viewing the trajectory and $\mathscr {M}_{s}$ in $(ca_{i}, c_{\mathrm{tot}}, l)$-space. To visualize this projection, we notice that during the long silent phase of the SS solution, *v* restricted to the surface $\hat{f}_{1}=0$ is approximately a constant denoted by $\bar{v}\approx-0.5$ (see Figs. 2C, 13B and 14A). $\hat{f}_{2}$ does not depend on *h*, so we can substitute $\bar{v}\approx-0.5$ and solve $\hat{f}_{2}(\bar{v}, c, c_{\mathrm{tot}}, l)=0$ for $c_{\mathrm{tot}}$ as a function of *c* and *l* and can therefore readily visualize the projection of $\mathscr {M}_{s}$ onto $(c, c_{\mathrm{tot}}, l)$-space (Fig. 14B). Also shown in Fig. 14A and B are the projections of the solution trajectory (black) and $\mathscr {M}_{ss}$ (red). As *v* jumps up to a larger range of values during the *v*-spike, the critical manifold $\mathscr {M}_{s}$ should no longer be represented in $(ca_{i}, c_{\mathrm{tot}}, l)$-space by the $ca_{i}$-nullsurface fixed at *v̄*. However, reasons similar to those discussed in Sect. 3, the calcium equation only depends weakly on *v* during the active phase; that is, as the trajectory enters the spiking phase, the $ca_{i}$-nullsurfaces for $v=\bar{v}$ and for $v=v_{\max}$ lie extremely close to each other. Therefore, it suffices to consider the single $ca_{i}$-nullsurface for $v=-0.5$ to qualitatively understand the projection of the full SS dynamics shown in Fig. 14B.

By having visualizations of $\mathscr {M}_{s}$ and $\mathscr {M}_{ss}$ projected onto both $(v, h, ca_{i})$-space and $(ca_{i}, c_{\mathrm{tot}}, l)$-space, we can now understand the evolution of the SS solution in terms of the shapes and relative positions of $\mathscr {M}_{s}$ and $\mathscr {M}_{ss}$. Starting from the yellow star in Fig. 14, the trajectory is in the silent phase and evolves on the slow timescale under the slow reduced layer problem (9a)–(9b), until it approaches sufficiently close to the superslow manifold $\mathscr {M}_{ss}$ (near the yellow circle). From there, the trajectory evolves on the superslow timescale under (10). It follows $\mathscr {M}_{ss}$ to a subcritical AH bifurcation (yellow diamond) of the fast-slow subsystem with respect to bifurcation parameter $c_{\mathrm{tot}}$, where a branch of unstable small amplitude periodic orbits is born (not shown here) and $\mathscr {M}_{ss}$ becomes unstable (yellow diamond). From there, the trajectory makes a fast jump to large *v* and $ca_{i}$, governed by (8a)–(8b); this jump corresponds to the onset of a spike in *v* in the SS solution. After the trajectory reaches $\mathscr {M}_{s}$ after the fast jump, the active phase begins. As we can see from Fig. 14B, $ca_{i}$ is relatively large near the right branch of the $ca_{i}$-nullsurface and therefore, as discussed previously, $c_{\mathrm{tot}}$ is no longer a superslow variable. Thus, during the spiking phase, the flow is governed by the slow reduced problem (11a)–(11c). Since there are no branches of $\mathscr {M}_{ss}$ on $\mathscr {M}_{s}$ for $ca_{i}$ large, the trajectory moves on the slow timescale along $\mathscr {M}_{s}$ under (11a)–(11c) until it meets the lower fold of the $ca_{i}$-nullsurface (Fig. 14B), from which it jumps back to its starting point (yellow star) on the fast timescale under (8a)–(8b), completing a full cycle.

**Fig. 15:**
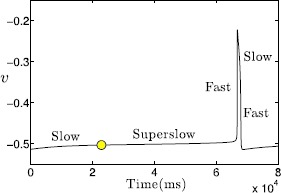
Simulation of the sigh-like spiking solution. Sigh-like spiking solution of (7a)–(7e). The *yellow symbol* indicates the transition point between slow and superslow flow

Based on the above analysis, we summarize the different timescales on which *v* evolves in Fig. 15, where the yellow circle indicates the transition point between the slow and superslow timescales that occurs as the trajectory reaches a small neighborhood of $\mathscr {M}_{ss}$ as it evolves along the lower-*v* surface of $\mathscr {M}_{s}$.

### Identifying Timescales

Next, we seek to identify whether the SS solution discussed above is truly a three-timescale phenomenon. In our original scaling, the Toporikova model has 2 fast, 2 slow and 1 superslow (2F, 2S, 1SS) variables. Similarly to Sect. 3.2, we assess the importance of having three timescales in two natural ways, by adjusting the two slow variables to be either fast or superslow. That is, we first speed up *h* and *l* by decreasing $\bar{\tau}_{h}$ and increasing *A* by a factor of 100, respectively, so that they evolve on the same timescale as *v* and $ca_{i}$, resulting in a 4 fast, 1 superslow (4F, 1SS) system. Second, we slow down *h* and *l* by adjusting $\bar{\tau}_{h}$ and *A* in the opposite way to produce a system with 2 fast and 3 superslow (2F, 3SS) variables. Then we consider whether or not these two-timescale systems can generate solutions that are similar to the SS solution.

**Fig. 16:**
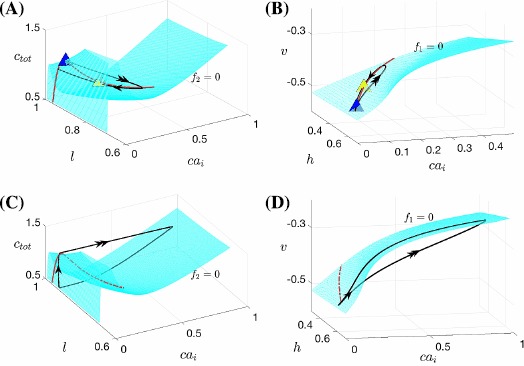
Simulations of the Toporikova model with two-time-scale reductions. Projections of solutions for the (4F, 1SS) system (*top*: **A**, **B**) and the (2F, 3SS) system (*bottom*: **C**, **D**) onto $(ca_{i}, c_{\mathrm{tot}}, l)$-space (*left column*) and $(v, h, ca_{i})$-space (*right column*). The surfaces represent different projections of $\mathscr {M}_{s}$, as shown in Fig. 14. *The red curve* denotes $\mathscr {M}_{ss}$ and *blue and yellow triangles* in (**A**), (**B**) mark two folds of $\mathscr {M}_{ss}$. Note that $\mathscr {M}_{s}$ is not relevant for the trajectory in (**A**), (**B**), nor is $\mathscr {M}_{ss}$ for the trajectory in (**C**), (**D**)

In the (4F, 1SS) rescaling, a different type of trajectory lacking large *v* and $ca_{i}$ spikes is observed (see Fig. 16A and B for different projections of the solution). For the fast layer dynamics of the (4F, 1SS) system, the critical manifold is our former superslow manifold, $\mathscr {M}_{ss}$, which lies within $\mathscr {M}_{s}$. The outer branches of $\mathscr {M}_{ss}$ are stable while the middle branch is unstable with respect to the layer system. The stable trajectory of the (4F, 1SS) system is attracted to a stable branch of $\mathscr {M}_{ss}$. Eventually, the trajectory passes the fold of $\mathscr {M}_{ss}$ (blue triangle) where $\mathscr {M}_{ss}$ destabilizes, a transition to the fast layer problem occurs, and hence the solution of the (4F, 1SS) system follows the fast layer flow to another stable branch of $\mathscr {M}_{ss}$. The subsequent motion is governed by the superslow flow until *v* and $ca_{i}$ jump down from another fold of $\mathscr {M}_{ss}$ (yellow triangle). As seen in Fig. 16, in the (4F, 1SS) scaling, *v*, $ca_{i}$ stay small throughout the oscillation between branches of $\mathscr {M}_{ss}$, and hence the large spikes are lost in the *v* and $ca_{i}$ time series.

Under the alternative rescaling to a (2F, 3SS) system, the projections of the solution trajectory are as shown in Fig. 16C and D. This system has the same critical manifold $\mathscr {M}_{s}$ as the original system. In contrast to the (4F, 1SS) system, $\mathscr {M}_{ss}$, although shown for comparison, is no longer meaningful in the (2F, 3SS) system. Hence, the trajectory simply follows $\mathscr {M}_{s}$, and a standard two-timescale relaxation oscillation results.

In summary, neither of these two-timescale systems captures the full features of the oscillating solution shown in Fig. 2C. Since a two-timescale system will lose either the large spikes in the *v* and $ca_{i}$ time series or the two-scale aspect of the recovery of *v* between spikes, we classify the SS solution as an intrinsically three-timescale phenomenon. We note, however, that the qualitative difference between the SS solution for the (2F, 2S, 1SS) and (2F, 3SS) scalings are not nearly as significant as the differences arising in the three- and two-timescale scalings for the SB model (1a)–(1g).

## Comparison of Results for SB and SS Solutions

We are now in a position to compare mechanisms underlying SB and SS solutions and how various timescales are manifested in the two solutions. At a general initial condition within the equilibrium set of the relevant fast subsystem (denoted by *S* in the Jasinski model and $\mathscr {M}_{s}$ in the Toporikova model, respectively), both SB and SS solutions evolve on a slow timescale. From such an initial condition, the solutions will converge to different dynamic regimes. Specifically, the SB solution is attracted by a stable bursting state for the fast-slow subsystem (as shown in Fig. 7), yielding multiple regular bursting cycles. In contrast, the SS solution converges to $\mathscr {M}_{ss}$ and hence enters the quiescent state, where a plateau of *v* results. Although constrained to different fast-slow subsystem attractors, both solution trajectories are governed by the dynamics of the superslow variables, variations of which will then push the fast-slow subsystem, which consists of all the fast and slow variables with superslow variables as static parameters, through some bifurcations at which either a sigh-like burst or a sigh-like spike can occur.

Despite this commonality, the mechanisms underlying the transitions from regular bursts to the sigh-like burst and from the quiescent plateau to the sigh-like spike differ in the two models. Specifically, for the SB solution, the sigh-like burst arises as the increase of superslow variables, $c_{\mathrm{tot}}$ and *l*, switches the behavior of the fast-slow subsystem from bursting to tonic spiking as the bursting branch loses stability at a SN bifurcation of periodic orbits ($\mathrm{SN}_{1}$ in Figs. 7 and 10C). In contrast, the sigh-like spike in the SS solution occurs as the trajectory crosses an AH bifurcation of the fast-slow subsystem of (7a)–(7e), where $\mathscr {M}_{ss}$ becomes unstable. Different bifurcations associated with the generation of sighs in the two models induce different requirements on the timescale of calcium to produce sighing activity. As discussed in Sect. 3.2, $ca_{i}$ cannot be grouped together with *v* as a fast variable to generate SB solutions. There is no such constraint on $ca_{i}$ in producing SS solutions, however. If an experimental manipulation could accelerate calcium dynamics, then the loss of sighing dynamics would support the SB model, which involves a unified mechanism for eupneic and sigh-like burst generation, whereas little change in sighing dynamics would support the SS model, which is based on separate eupneic and sigh-like burst generation mechanisms.

Another difference between SB and SS solutions lies in the transition of $ca_{i}$ to large values. In the SS solution, $ca_{i}$ jumps up after the AH bifurcation on $\mathscr {M}_{ss}$, which is the same bifurcation inducing the sigh-like spike. The jump-up of $ca_{i}$ in the SB solution, however, happens at a different bifurcation than that for the onset of a sigh-like burst. After an additional drift on the superslow timescale, the transition occurs at a SN bifurcation of a spiking branch of the fast-slow subsystem of the Jasinski model ($\mathrm{SN}_{2}$ in Figs. 7 and 10). In other words, the observation that a surge in calcium coincides with the onset of sighing would support the SS model, whereas a finding that the surge in calcium occurs after sighing is already under way would support the SB model.

After the rapid increase of $ca_{i}$, the trajectory enters the active phase, during which the SB model generates continuous spiking constituting the sigh-like burst, which is still governed by the dynamics of superslow variables (i.e., $c_{\mathrm{tot}}$ and *l*). In the SS model, during the active phase when the big spike occurs, the evolution of the solution along the right branch of the calcium nullsurface is governed by the slow reduced problem and hence occurs on a slow timescale, such that the SS sigh event has a shorter duration than the sigh event in the SB solution.

Finally, termination mechanisms for both SB and SS solutions involve $ca_{i}$-dependent inactivation of $[\mathrm{IP}_{3}]$ (regulated by *l*), resulting in the reduction of $ca_{i}$ and deactivation of $I_{\mathrm{CAN}}$ [13]. In both SB and SS solutions, regular oscillations (bursting/spiking) persist when $I_{\mathrm{CAN}}$ is removed, whereas the sigh-like oscillations are eliminated under the supression of $I_{\mathrm{CAN}}$ or $I_{\mathrm{Ca}}$, confirming that the sigh-like activities in both models critically depend on both $I_{\mathrm{CAN}}$ and $I_{\mathrm{Ca}}$.

## Discussion

To understand the mechanisms underlying the generation of sighs, we considered two distinct single-compartment models for respiratory neurons in the pre-BötC that have the ability to generate sigh-like activity. For both models, we performed nondimensionalization to identify possible groupings of variables into classes corresponding to they timescales on which they evolve. After establishing such groupings, in some cases with class membership changing depending on the location of the trajectory in phase space, we used a non-rigorous GSPT approach to elucidate the roles of various timescale-based subsystems and their bifurcation structures in producing sigh-like dynamics. We identified commonalities and differences between the mechanisms involved for the two models and argued that for both models, the sigh-like dynamics involves three timescales in an essential way. This work adds to the growing literature of dynamical systems analyses of three-timescale systems [15, 23, 25–30] and in particular to recent efforts to identify how many timescales are really needed to produce particular dynamic patterns [15, 23]. It also provides information about model parameter relationships needed to support sigh-like activity, which may be useful for future efforts to model the repertoire of pre-BötC dynamics and their variations under normal conditions, environmental and metabolic challenges, and pathologies [2].

The first model that we considered is a self-coupled neuron model featuring $I_{\mathrm{NaP}}$, $I_{\mathrm{CAN}}$ and the $\mathrm{Na}^{+}/\mathrm{K}^{+}$-pump current. An aspect of this model that is more biologically realistic than previous models studied in [15] and [23] is the inclusion of bidirectional coupling between voltage and calcium dynamics. In Sect. 3, we extended and applied analysis methods from [15] to the Jasinski model (1a)–(1g) and explained the mechanisms underlying its SB solutions. While the bidirectional coupling between *V* and $\mathrm{Ca}_{\mathrm{i}}$ as well as more detailed $\mathrm{Ca}^{2+}$ dynamics make the implementation of the decomposition method more difficult than in past work on similar models, fast-slow averaging allowed us to complete the analysis. Besides describing specific details of the SB solution features, we have also investigated whether this solution fundamentally involves three timescales. Unlike the MB solution in [13–15], our analysis shows that SB solution features are lost under the natural groupings to two timescales, supporting the preliminary conclusion that SB dynamics in system (1a)–(1g) requires at least three timescales. A more rigorous demonstration of this requirement is still an open matter, and indeed rigorous proofs that particular solution types can only occur when three (or more) timescales are present have not, to our knowledge, been provided in the literature to date.

Several conditions that support the existence of the SB solutions can also be deduced from our analysis. To obtain SB solutions, we require relatively small $g_{\mathrm{Ca}}$, whereas large $g_{\mathrm{Ca}}$ will eliminate SB patterns in the Jasinski model. That is, the increase of $g_{\mathrm{Ca}}$ will speed up $\mathrm{Ca}_{\mathrm{i}}$ and $\mathrm{Ca}_{\mathrm{tot}}$ (see Table 5) such that the time available for the regular bursting phase becomes shorter and as a result, the number of small bursts decreases. With further increases in $g_{\mathrm{Ca}}$, the regular bursts will completely disappear and the SB solution will be lost. Second, the regular bursting phase also necessitates a long enough time before $\mathrm{Ca}_{\mathrm{i}}$ jumps up to allow the solution trajectory to undergo multiple crossings between the LF and HC curves in the slow $(\mathrm{Na}_{\mathrm{i}}, \mathrm{Ca}_{\mathrm{i}})$-space. As suggested by the (9F, 2SS) case shown in Fig. 11, each crossing between the LF and HC curves should also be slow enough for the full system to generate a burst, rather than simply a spike. On the other hand, if the evolution of $\mathrm{Na}_{\mathrm{i}}$ and $\mathrm{Ca}_{\mathrm{i}}$ are too slow for the solution to complete a single square-wave burst before the fast subsystem transitions to tonic spiking due to the evolution of $\mathrm{Ca}_{\mathrm{tot}}$ and *l*, as shown in Fig. 12, the SB solution no longer exists. This suggests that the existence of the SB solution requires the timescale for $\mathrm{Ca}_{\mathrm{i}}$, $\mathrm{Na}_{\mathrm{i}}$ to be faster than $\mathrm{Ca}_{\mathrm{tot}}$, *l*. These types of arguments could be useful for deriving a minimal biological model for SB dynamics, although we do not complete this step here since we do not have a particular application in mind for such a model. Similar analysis can also been used to adjust parameters in order to enhance the robustness of SB solutions with respect to other parameter variations, as has been done for another multiple-timescale respiratory neuron model [15].

Another possible direction for future work is to explain why the self-excitation in system (1a)–(1g) appears to be required for the SB solution to exist. In our setting, we have chosen $g_{\mathrm{SynE}}=20$ to obtain the SB solutions and a sufficient decrease of $g_{\mathrm{SynE}}$ will eliminate the SB dynamics, which agrees with the findings from [8] that SB behavior requires excitatory synaptic inputs. A preliminary numerical simulation shows that a decrease of $g_{\mathrm{SynE}}$ can result in a transition from bursting to spiking in the voltage compartment, preventing the regular square-wave bursting phase from occurring at low $\mathrm{Ca}_{\mathrm{i}}$ as needed for SB dynamics.

In addition to the Jasinski model, we have also considered a second model for inspiratory pre-BötC neurons (3a)–(3e), which can yield SS solutions [9]. Similarly as for the Jasinski model, we applied GPST to understand the dynamics of the SS solution. By doing so, we discovered that the SS solution is not just a simple reduction of the SB solution in the same way that a relaxation oscillator can be viewed as a simplification of a square-wave burster. Instead, the models differ in terms of the mechanisms underlying their high-amplitude sigh-like activities as well as in the details of which timescales control particular solution features, as discussed in Sect. 5. Nonetheless, sigh-like activities in both models depend critically on calcium oscillations, consistent with the previous experimental data showing that $I_{\mathrm{Ca}}$ blocker and $I_{\mathrm{CAN}}$ blocker [6, 7, 24] could terminate sighs generated in medullary slices containing the pre-BötC without suppressing regular bursting activity.

As with the SB solution, we also demonstrate that SS dynamics appears to be a three-timescale behavior. In addition, an interesting mathematical problem arising from the analysis of the SS solution is the development of systematic methods to treat equations that evolve on different timescales in different parts of phase space (cf. [31]). In Sect. 4, we have illustrated in a non-rigorous way how the GPST approach can be extended to a multiple-timescale model where the timescale of one variable, $\mathrm{Ca}_{\mathrm{tot}}$, changes with respect to the location of a trajectory in phase space. Moreover, according to the nondimensionalization results in Appendix 2, we find that the calcium derivative, denoted by $f_{2}$, can also be represented as a combination of functions with different relative sizes. Therefore, in different parts of phase space, $\mathrm{Ca}_{\mathrm{i}}$ can evolve on the fast, slow or superslow timescale. We repeated our GSPT analysis in a way that takes this observation into consideration, but no qualitatively additional information was gained from doing so. Hence, $\mathrm{Ca}_{\mathrm{i}}$ is simply treated as a fast variable in our analysis of SS dynamics in this paper. Development of a more systematic and rigorous approach for assessing when phase-dependent scalings are important would be a helpful step for future analyses.

## Appendix 1: Nondimensionalization of the Jasinski Model

Numerical simulations of system (1a)–(1g) show that the membrane potential *V* lies between $-60~\mbox{mV}$ and $20~\mbox{mV}$ (Fig. 3). $T_{y}$ is defined to be $\max(1/\tau_{y}(V))$ over the range $V\in[-60, 20]$ and hence we obtain $T_{m_{\mathrm{Na}}}\approx190.64~\mbox{ms}^{-1}$, $T_{h_{\mathrm{Na}}}\approx55~\mbox{ms}^{-1}$, $T_{m_{\mathrm{Ca}}}\approx2~\mbox{ms}^{-1}$, $T_{h_{\mathrm{Ca}}}\approx0.056~\mbox{ms}^{-1}$, $T_{m_{\mathrm{K}}}\approx0.67~\mbox{ms}^{-1}$ and $T_{s}\approx0.2~\mbox{ms}^{-1}$, respectively. $g_{\max}$, the maximum of all conductances, is $160~\mbox{nS}$. Substituting these expressions into system (1a)–(1g) and rearranging, we obtain the dimensionless version of the 11-dimensional neuron model:

12a $$\begin{aligned} \frac{C}{Q_{t}\cdot g_{\max}}\frac{dv}{d\tau} =&-\bar{I}_{\mathrm {Na}}- \bar{I}_{\mathrm{NaP}}-\bar{I}_{\mathrm{K}}-\bar{I}_{\mathrm {Ca}}- \bar{I}_{\mathrm{CAN}} \\ &{}-\bar{I}_{\mathrm{Pump}}-\bar{I}_{\mathrm {L}}- \bar{I}_{\mathrm{SynE}}, \end{aligned}$$

12b $$\begin{aligned} \frac{1}{Q_{t}\cdot T_{y}}\frac{dy}{d\tau} =& \bigl(y_{\infty}(v)-y \bigr)/{t_{y}(v)}, \end{aligned}$$

12c $$\begin{aligned} \frac{Q_{c}}{Q_{t}\cdot\alpha_{\mathrm{Ca}}\cdot g_{\mathrm{Ca}}\cdot Q_{v}}\frac {d c_{\mathrm{tot}}}{d\tau} =&-\frac{I_{\mathrm{Ca}}}{g_{\mathrm{Ca}} Q_{v}}-\frac {ca_{i}}{K' _{c_{\mathrm{tot}}}}, \end{aligned}$$

12d $$\begin{aligned} \frac{Q_{c}}{Q_{t}\cdot\alpha_{\mathrm{Ca}}\cdot g_{\mathrm{Ca}}\cdot Q_{v}}\frac {d ca_{i}}{d\tau} =& -\frac{I_{\mathrm{Ca}}}{g_{\mathrm{Ca}} Q_{v}}-\frac {ca_{i}}{K'_{c_{\mathrm{tot}}}}+ K'_{c}(\bar{J}_{\mathrm{ER}_{\mathrm{IN}}}-\bar{J}_{\mathrm{ER}_{\mathrm{OUT}}}), \end{aligned}$$

12e $$\begin{aligned} \frac{1}{Q_{t}\cdot Q_{c}\cdot A}\frac{dl}{d\tau} =& \bar{K}_{\mathrm{d}}(1-l)-ca_{i} l, \end{aligned}$$

12f $$\begin{aligned} \frac{Q_{na}}{Q_{t}\cdot\alpha_{\mathrm{Na}}\cdot g_{\max}\cdot Q_{v}}\frac{d na_{i}}{d\tau} =& -(\bar{I}_{\mathrm{Na}}+ \bar{I}_{\mathrm {NaP}}+\bar{I}_{\mathrm{CAN}}+3\bar{I}_{\mathrm{Pump}}), \end{aligned}$$

12g $$\begin{aligned} \frac{1}{Q_{t}\cdot T_{s}}\frac{ds}{d\tau} =&\bigl((1-s) s_{\infty }(V)-s \bigr)/t_{s}(V), \end{aligned}$$

where $y=\{m_{\mathrm{Na}}, h_{\mathrm{Na}}, m_{\mathrm{Ca}}, h_{\mathrm {Ca}}, m_{\mathrm{K}}\}$.

The dimensionless currents $\bar{I}_{x}$ appearing in (12a) and (12f) are given by $\frac {I_{x}}{g_{\max}Q_{v}}$. Dimensionless calcium fluxes are given by $\bar{J}_{\mathrm{ER}_{\mathrm{IN}}}=J_{\mathrm{ER}_{\mathrm{IN}}} \sigma/P_{\max}$ and $\bar{J}_{ \mathrm{ER}_{\mathrm{OUT}}}=J_{\mathrm{ER}_{\mathrm{OUT}}} \sigma/P_{\max}$, where $P_{\max}$ takes the value $1\text{,}148$ according to numerical results. In Eqs. (12c) and (12d), $K'_{c_{\mathrm{tot}}}=\tau_{\mathrm{Ca}}\cdot\alpha_{\mathrm{Ca}}\cdot g_{\mathrm{Ca}}\cdot Q_{v}/Q_{c}$ and $K'_{c}=\frac{\mathrm{K}_{\mathrm {C}a}\cdot P_{\max}}{\alpha_{\mathrm {Ca}}\cdot\sigma\cdot g_{\mathrm{Ca}}\cdot Q_{v}}$.

Since we expect $V \in[-60, 20]$, a suitable choice for the voltage scale is $Q_{v}=100~\mbox{mV}$. Similarly, we choose $Q_{na}=30~\mbox{mM}$ since $\mathrm{Na}_{\mathrm{i}}\in[16, 21]$. $Q_{c}$, the scale for $\mathrm{Ca}_{\mathrm{i}}$ and $\mathrm{Ca}_{\mathrm{tot}}$, should be determined by magnitudes of both variables. Numerical simulations show that $\mathrm{Ca}_{\mathrm{i}}$ varies from $O(10^{-5})$ to $O(10^{-3})$, while $\mathrm{Ca}_{\mathrm{tot}}$ is roughly $O(10^{-3})$ (Fig. 4B and C). Therefore, we choose $Q_{c}=10^{-3}~\mbox{mM}$. Using these scales, we see that all terms in the right-hand sides of Eqs. (12a)–(12g) are bounded (in absolute value) by one except that the last term in (12d) is roughly $O(10)$. To resolve this issue, we divide both sides of (12d) by 10 and obtain the following:

13 $$\begin{aligned} &\frac{Q_{c}}{10 Q_{t}\cdot\alpha_{\mathrm{Ca}}\cdot g_{\mathrm{Ca}}\cdot Q_{v}}\frac {d ca_{i}}{d\tau} \\ &\quad = - \frac{I_{\mathrm{Ca}}}{10 g_{\mathrm{Ca}} Q_{v}}-\frac {ca_{i}}{10 K_{c_{\mathrm{tot}}}}+ \frac{K_{c}}{10}(\bar{J}_{\mathrm{ER}_{\mathrm{IN}}}- \bar {J}_{\mathrm{ER}_{\mathrm{OUT}}}), \end{aligned}$$

the right-hand side of which is now $O(1)$, as we desire for our nondimensionalization.

The coefficients of the derivatives in the left-hand sides of Eqs. (12a)–(12g) with (12d) replaced by (13) now reveal the relative speeds of evolution of the variables. We summarize the timescales for variables of the Jasinski model in Table 5.

**Table 5:** **The timescales for the Jasinski model (** **1a** **)–(** **1g** **)**

Variable	Coefficient of the derivative
*V*	$C/g_{\max} \approx0.225~\mbox{ms}$
$m_{\mathrm{Na}}$	$\frac{1}{T_{y}}\approx0.0053~\mbox{ms}$
$h_{\mathrm{Na}}$	$\frac{1}{T_{y}} \approx0.0182~\mbox{ms}$
$m_{\mathrm{Ca}}$	$\frac{1}{T_{y}} \approx0.5~\mbox{ms}$
$h_{\mathrm{Ca}}$	$\frac{1}{T_{y}} \approx18~\mbox{ms}$
$m_{K}$	$\frac{1}{T_{y}} \approx1.4925~\mbox{ms}$
*s*	$\frac{1}{T_{y}} \approx5~\mbox{ms}$
$\mathrm{Ca}_{\mathrm{i}}$	$Q_{c}/(10\alpha_{\mathrm{Ca}}\cdot g_{\mathrm {Ca}}\cdot Q_{v})\approx61.5~\mbox{ms}$
$\mathrm{Na}_{\mathrm{i}}$	$Q_{na}/(\alpha_{\mathrm{Na}}\cdot g_{\max}\cdot Q_{v}) \approx37.5~\mbox{ms}$
$\mathrm{Ca}_{\mathrm{tot}}$	$Q_{c}/(\alpha_{\mathrm{Ca}}\cdot g_{\mathrm {Ca}}\cdot Q_{v}) \approx615.3846~\mbox{ms}$
*l*	$1/( Q_{c}\cdot A) \approx10\text{,}000~\mbox{ms}$

Comparing the values of these coefficients indicates how fast each corresponding variable is; the larger the value, the slower the corresponding variable. According to our nondimensionalization results summarized in Table 5, we choose to group together *V*, the gating variables $m_{\mathrm{Na}}$, $h_{\mathrm{Na}}$, $m_{\mathrm{Ca}}$, $h_{\mathrm{Ca}}$, $m_{\mathrm{K}}$, and *s* as the variables evolving on a fast timescale. We group $(\mathrm{Ca}_{\mathrm{i}}, \mathrm{Na}_{\mathrm{i}})$ as evolving on a slow timescale and $(\mathrm{Ca}_{\mathrm{tot}}, l)$ as evolving on a superslow timescale. We choose the slow timescale as our reference time, i.e., pick $Q_{t}=100~\mbox{ms}$ and let $R_{x}$ denote coefficients of $\frac{dx}{dt}$ in equations (12a)–(12b), (13) and (12e)–(12g), the dimensionless system then becomes the system (5a)–(5g) given in Sect. 3.1, namely,

14a $$\begin{aligned} R_{v}\frac{dv}{d\tau} =&-\bar{I}_{\mathrm{Na}}- \bar{I}_{\mathrm{NaP}}-\bar {I}_{\mathrm{K}}-\bar{I}_{\mathrm{Ca}}- \bar{I}_{\mathrm{CAN}}-\bar {I}_{\mathrm{Pump}}-\bar{I}_{\mathrm{L}}- \bar{I}_{\mathrm{SynE}} \\ :=& f(v,y,s,ca_{i},na_{i}), \end{aligned}$$

14b $$\begin{aligned} R_{y}\frac{dy}{d\tau} =&\bigl(y_{\infty}(v)-y \bigr)/{t_{y}(v)}:=H(v,y), \end{aligned}$$

14c $$\begin{aligned} R_{c_{\mathrm{tot}}}\frac{d c_{\mathrm{tot}}}{d\tau} =&-\frac{I_{\mathrm {Ca}}}{g_{\mathrm{Ca}} Q_{v}}-\frac {ca_{i}}{K'_{c_{\mathrm{tot}}}}:=h_{1}(v,ca_{i}), \end{aligned}$$

14d $$\begin{aligned} R_{ca_{i}}\frac{d ca_{i}}{d\tau} =& -\frac{I_{\mathrm{Ca}}}{g_{\mathrm{Ca}} Q_{v}}-\frac{ca_{i}}{K'_{c_{\mathrm{tot}}}}+ K'_{c}(\bar{J}_{\mathrm{ER}_{\mathrm{IN}}}-\bar {J}_{\mathrm{ER}_{\mathrm{OUT}}}) \\ :=&g_{1}(v,ca_{i},c_{\mathrm{tot}},l), \end{aligned}$$

14e $$\begin{aligned} R_{l}\frac{dl}{d\tau} =& \bar{K}_{\mathrm{d}}(1-l)-ca_{i} l:=h_{2}(ca_{i},l), \end{aligned}$$

14f $$\begin{aligned} R_{na_{i}}\frac{d na_{i}}{d\tau} =& -(\bar{I}_{\mathrm{Na}}+ \bar{I}_{\mathrm {NaP}}+\bar{I}_{\mathrm{CAN}}+3\bar{I}_{\mathrm{Pump}}):=g_{2}(v,na_{i},ca_{i}), \end{aligned}$$

14g $$\begin{aligned} R_{s}\frac{ds}{d\tau} =&\bigl((1-s) s_{\infty}(V)-s \bigr)/t_{s}(V):=S(v,s). \end{aligned}$$

## Appendix 2: Nondimensionalization of the Toporikova Model

From numerical simulations of system (3a)–(3e), we find that the membrane potential *V* typically lies between $-60~\mbox{mV}$ and $20~\mbox{mV}$. Correspondingly, we define $T_{h}=\max(1/\tau_{h}(V))$ over the range $V\in[-60, -20]$ and then define $t_{h}(V)$, a rescaled version of $\tau_{h}(V)$, by $t_{h}(V)=T_{h}\tau_{h}(V)$. We also define $g_{\max}$ to be the maximum of the five conductances $g_{\mathrm {NaP}}$, $g_{\mathrm{K}}$, $g_{\mathrm{Ca}}$, $g_{\mathrm{CAN}}$, $g_{\mathrm{h}}$. Furthermore, we let $G(\mathrm{Ca}_{\mathrm{i}}):=\frac{[\mathrm{IP}_{3}]\mathrm{Ca}_{\mathrm{i}}}{([\mathrm{IP}_{3}+K_{I}])(\mathrm{Ca}_{\mathrm{i}}+K_{a})}$, $g_{\mathrm{SERCA}}(\mathrm{Ca}_{\mathrm{i}}):=\frac {V_{\mathrm{SERCA}}\mathrm{Ca}_{\mathrm{i}}}{K^{2}_{\mathrm{SERCA}}+\mathrm{Ca}^{2}_{\mathrm{i}}}$ and $\phi(\mathrm{Ca}_{\mathrm{i}}):=\frac{V_{\mathrm{PMCA}}\mathrm{Ca}_{\mathrm{i}}}{K^{2}_{\mathrm{PMCA}}+\mathrm{Ca}^{2}_{\mathrm{i}}}$. Then we have $J_{\mathrm{ER}_{\mathrm{IN}}}=(L_{\mathrm{IP}_{3}}+P_{\mathrm{IP}_{3}} G^{3}(\mathrm{Ca}_{\mathrm{i}}) l^{3})(\mathrm{Ca}_{\mathrm{ER}}-\mathrm{Ca}_{\mathrm{i}})$ and $J_{\mathrm{ER}_{\mathrm{OUT}}}=g_{\mathrm{SERCA}}(\mathrm{Ca}_{\mathrm{i}})\mathrm{Ca}_{\mathrm{i}}$, respectively. Substituting these expressions into Eqs. (3a)–(3e) and rearranging, we obtain the following system:

15a $$\begin{aligned} \frac{C_{m}}{Q_{t}\cdot g_{\max}}\frac{dv}{d\tau} =&-\bar{I}_{\mathrm {NaP}}+ \bar{I}_{\mathrm{leak}}-\bar{I}_{\mathrm{Ca}}-\bar{I}_{\mathrm {CAN}}-\bar {I}_{h}, \end{aligned}$$

15b $$\begin{aligned} \frac{1}{Q_{t}\cdot T_{h} }\frac{dh}{d\tau} =&\bigl(h_{\infty }(v)-h \bigr)/{t_{h}(v)}, \end{aligned}$$

15c $$\begin{aligned} \frac{d\mathrm{Ca}_{\mathrm{tot}}}{dt} =&\frac{\mathrm{K}_{\mathrm{Ca}}}{\lambda} \bigl(-\alpha _{\mathrm{Ca}} I_{\mathrm{Ca}}-\phi(\mathrm{Ca}_{\mathrm{i}}) \mathrm{Ca}_{\mathrm{i}} \bigr), \end{aligned}$$

15d $$\begin{aligned} \frac{d[\mathrm{Ca}]}{dt} =& \mathrm{K}_{\mathrm{Ca}} (J_{\mathrm{ER}_{\mathrm{IN}}}-J_{\mathrm{ER}_{\mathrm{OUT}}} )+ \frac{\mathrm{K}_{\mathrm{Ca}}}{\lambda} \bigl(-\alpha_{\mathrm {Ca}}I_{\mathrm{Ca}}-\phi( \mathrm{Ca}_{\mathrm{i}}) \mathrm{Ca}_{\mathrm{i}} \bigr), \end{aligned}$$

15e $$\begin{aligned} \frac{dl}{dt} =& AK_{\mathrm{d}}(1-l)-A[\mathrm{Ca}] l, \end{aligned}$$

with dimensionless currents $\bar{I}_{x}=\frac{I_{x}}{g_{\max}Q_{v}}$. We have now nondimensionalized the equations for *V* and *h*, and next we deal with equations for the calcium compartment (15c)–(15e). From numerical simulations, $\mathrm{Ca}_{\mathrm{i}}$ and $\mathrm{Ca}_{\mathrm{tot}}$ typically lie between $0~\mu\mbox{M}$ and $1~\mu\mbox{M}$. Therefore, we define $G_{c}=\max(G^{3}(\mathrm{Ca}_{\mathrm{i}}))$, $G_{S}=\max(g_{\mathrm{SERCA}}(\mathrm{Ca}_{\mathrm{i}}))$ and $\varPhi =\max(\phi(\mathrm{Ca}_{\mathrm{i}}))$ over the range $\mathrm{Ca}_{\mathrm{i}} \in[0, 1]$ and define $P_{\max}$ to be the maximum of $\{L_{\mathrm{IP}_{3}}, P_{\mathrm{IP}_{3}}G_{c}, G_{S}\}$. From system (15a)–(15e), we obtain the following dimensionless system:

16a $$\begin{aligned} \frac{C_{m}}{Q_{t}\cdot g_{\max}}\frac{dv}{d\tau} =&-\bar{I}_{\mathrm {NaP}}+ \bar{I}_{\mathrm{leak}}-\bar{I}_{\mathrm{Ca}}-\bar{I}_{\mathrm {CAN}}-\bar {I}_{h}, \end{aligned}$$

16b $$\begin{aligned} \frac{1}{Q_{t}\cdot T_{h} }\frac{dh}{d\tau} =&\bigl(h_{\infty }(v)-h \bigr)/{t_{h}(v)}, \end{aligned}$$

16c $$\begin{aligned} \frac{Q_{c}\cdot\lambda}{Q_{t}\cdot \mathrm{K}_{\mathrm{Ca}}\cdot\alpha_{\mathrm {Ca}}\cdot g_{\mathrm{Ca}}\cdot Q_{v}}\frac{d c_{\mathrm{tot}}}{d\tau} =& \biggl(-\tilde {I}_{\mathrm{Ca}}- K_{c_{\mathrm{tot}}}\bar{\phi}(ca_{i})\frac{\mathrm{Ca}_{\mathrm{i}}}{Q_{c}} \biggr), \end{aligned}$$

16d $$\begin{aligned} \frac{\lambda}{Q_{t}\cdot \mathrm{K}_{\mathrm{Ca}}\cdot \alpha_{\mathrm{Ca}}\cdot g_{\mathrm{Ca}}\cdot Q_{v}}\frac{dca_{i}}{d\tau} =& K_{c} (\bar{J}_{\mathrm{ER}_{\mathrm{IN}}}- \bar{J}_{\mathrm{ER}_{\mathrm{OUT}}} ) \\ &{}+ \biggl(-\tilde{I}_{\mathrm{Ca}}- K_{c_{\mathrm{tot}}}\bar{ \phi}(ca_{i}) \frac{Ca_{i}}{Q_{c}} \biggr), \end{aligned}$$

16e $$\begin{aligned} \frac{1}{Q_{t}\cdot Q_{c} A}\frac{dl}{d\tau} =& \bar{K}_{\mathrm{d}}(1-l)-ca_{i} l, \end{aligned}$$

where $\tilde{I}_{\mathrm{Ca}}=\frac{I_{\mathrm{Ca}}}{g_{\mathrm{Ca}}Q_{v}}$, $K_{c_{\mathrm{tot}}}=\frac{Q_{c}\cdot\varPhi}{\alpha_{\mathrm{Ca}}\cdot g_{\mathrm {Ca}}\cdot Q_{v}}$, $\bar{\phi}(ca_{i})=\frac{\phi(Q_{c}\cdot ca_{i})}{\varPhi }$, $K_{c}=\frac{\lambda P_{\max}}{\alpha_{\mathrm{Ca}}\cdot\sigma\cdot g_{\mathrm{Ca}}\cdot Q_{v}}$, $\bar{J}_{\mathrm{ER}_{\mathrm{IN}}}=J_{\mathrm{ER}_{\mathrm{IN}}} \sigma /P_{\max}$, $\bar{J}_{\mathrm{ER}_{\mathrm{OUT}}}=J_{\mathrm{ER}_{\mathrm{OUT}}}\sigma/P_{\max}$ and $\bar{K}_{\mathrm{d}}=K_{\mathrm{d}}/Q_{c}$.

Since we expect $V\in[-60, -20]$ and $\mathrm{Ca}_{\mathrm{tot}}, \mathrm{Ca}_{\mathrm{i}} \in[0, 1]$, suitable choices for the voltage and calcium scales are $Q_{v}=100~\mbox{mV}$ and $Q_{c}=1~\mu\mbox{M}$, respectively. We also see that values of $h_{\infty}$, *h* and *l* all lie in the range $[0, 1]$. For the choice of parameters specified in Table 4, the maximum conductance is $g_{\mathrm{K}}$, so we have $g_{\max}=g_{\mathrm{K}}$. Numerical evaluation of $1/\tau_{h}(V)$ for $V\in[-60, -20]$ shows that $T_{h}\approx8\times10^{-4}~\mbox{ms}^{-1}=O(10^{-3})~\mbox{ms}^{-1}$. We also obtain $G_{c}\approx0.0456$ and $G_{S}\approx1\text{,}000~\mbox{pL}\cdot \mbox{ms}^{-1}$, so we have $P_{\max}\approx1412~\mbox{pL}\cdot \mbox{ms}^{-1}$ and hence $K_{c}\approx7\times10^{3}=O(10^{4})$. Similarly, we have $K_{c_{\mathrm{tot}}}\approx30$. Using these values, we see that all terms on the right-hand sides of (16a)–(16e) are bounded (in absolute value) by one except $K_{c_{\mathrm{tot}}}$ and $K_{c}$. To resolve these issues, we divide both sides of (16c) and (16d) by $K_{c_{\mathrm{tot}}}$ and $K_{c}$, respectively, and keep other equations unchanged from (16a)–(16e):

17a $$\begin{aligned} \frac{C_{m}}{Q_{t}\cdot g_{\max}}\frac{dv}{d\tau} =&-\bar{I}_{\mathrm {NaP}}+ \bar{I}_{\mathrm{leak}}-\bar{I}_{\mathrm{Ca}}-\bar{I}_{\mathrm {CAN}}-\bar {I}_{h}, \end{aligned}$$

17b $$\begin{aligned} \frac{1}{Q_{t}\cdot T_{h} }\frac{dh}{d\tau} =&\bigl(h_{\infty }(v)-h \bigr)/{t_{h}(v)}, \end{aligned}$$

17c $$\begin{aligned} \frac{Q_{c}\cdot\lambda}{Q_{t}\cdot \mathrm{K}_{\mathrm{Ca}}\cdot\alpha_{\mathrm {Ca}}\cdot g_{\mathrm{Ca}}\cdot Q_{v}\cdot K_{c_{\mathrm{tot}}}}\frac{d c_{\mathrm{tot}}}{d\tau } =& \biggl(-\frac{1}{K_{c_{\mathrm{tot}}}} \tilde{I}_{\mathrm{Ca}}- \bar{\phi }(ca_{i}) \frac{Ca_{i}}{Q_{c}} \biggr), \end{aligned}$$

17d $$\begin{aligned} \frac{\lambda}{Q_{t}\cdot \mathrm{K}_{\mathrm{Ca}}\cdot\alpha_{\mathrm{Ca}}\cdot g_{\mathrm{Ca}}\cdot Q_{v}\cdot K_{c}}\frac{dca_{i}}{d\tau} =& (\bar {J}_{\mathrm{ER}_{\mathrm{IN}}}- \bar{J}_{\mathrm{ER}_{\mathrm{OUT}}} ) \\ &{}+ \biggl(-\hat{I}_{\mathrm{Ca}}- \bar {K}_{c_{\mathrm{tot}}} \bar{\phi}(ca_{i}) \frac{Ca_{i}}{Q_{c}} \biggr), \end{aligned}$$

17e $$\begin{aligned} \frac{1}{Q_{t}\cdot Q_{c}\cdot A}\frac{dl}{d\tau} =& \bar{K}_{\mathrm{d}}(1-l)-ca_{i} l, \end{aligned}$$

where $\hat{I}_{\mathrm{Ca}}=\tilde{I}_{\mathrm{Ca}}/K_{c}$ and $\bar {K}_{c_{\mathrm{tot}}}=K_{c_{\mathrm{tot}}}/K_{c}$. Now all terms on the right-hand side of (17a)–(17e) are also bounded by 1. Since $K_{c}\gg1$ appears in the denominator of both subtracted terms in $(-\hat{I}_{\mathrm{Ca}}- \bar{K}_{c_{\mathrm{tot}}}\bar{\phi}(ca_{i})\frac {Ca_{i}}{Q_{c}})$, this expression can be treated as negligible compared with the other terms on the right-hand side of (17d). Therefore the coefficients of the derivatives on the left-hand sides of (17a)–(17e) now reveal the relative rates of evolution of the variables. We find that $C_{m}/g_{\max}=O(10)~\mbox{ms}$, $1/T_{h}=O(10^{3})~\mbox{ms}$, $Q_{c}\lambda/(\mathrm{K}_{\mathrm{Ca}}\alpha_{\mathrm {Ca}}g_{\mathrm{Ca}}Q_{v}K_{c_{\mathrm{tot}}})=O(10^{3})~\mbox{ms}$, $Q_{c}\lambda /(\mathrm{K}_{\mathrm{Ca}}\alpha _{\mathrm{Ca}}g_{\mathrm{Ca}}Q_{v}K_{c})=O(10)~\mbox{ms}$, and $1/(Q_{c}A)=O(10^{3})~\mbox{ms}$, from which we conclude that *v* and $ca_{i}$ evolve on a relatively fast timescale, while *h*, $c_{\mathrm{tot}}$ and *l* evolve on a relatively slow timescale. We choose the slow timescale as our reference time, i.e., pick $Q_{t}=1\text{,}000~\mbox{ms}$, and set

18a $$\begin{aligned} \varepsilon :=&\frac{C_{m}}{Q_{t}\cdot g_{\max}}\approx\frac{Q_{t}\cdot \mathrm{K}_{\mathrm{Ca}}\cdot\alpha_{\mathrm{Ca}}\cdot g_{\mathrm{Ca}}\cdot Q_{v}\cdot K_{c}}{Q_{c}\lambda}\ll1,\qquad R_{h}:=Q_{t}T_{h}, \end{aligned}$$

18b $$\begin{aligned} R_{c_{\mathrm{tot}}} :=&\frac{Q_{t}\cdot \mathrm{K}_{\mathrm{Ca}}\cdot\alpha_{\mathrm {Ca}}\cdot g_{\mathrm{Ca}}\cdot Q_{v}\cdot K_{c_{\mathrm{tot}}}}{Q_{c}\lambda},\qquad R_{l}:=Q_{t} Q_{c} A. \end{aligned}$$

Substituting expressions in (18a)–(18b) into (17a)–(17e) and rearranging, we obtain the following:

19a $$\begin{aligned} \varepsilon\frac{dv}{d\tau} =&-\bar{I}_{\mathrm{NaP}}+\bar{I}_{\mathrm{leak}}- \bar{I}_{\mathrm{Ca}}-\bar{I}_{\mathrm{CAN}}-\bar{I}_{h}:=\hat {f}_{1}(v,h,ca_{i}), \end{aligned}$$

19b $$\begin{aligned} \frac{dh}{d\tau} =&R_{h} \bigl(h_{\infty}(v)-h \bigr)/{t_{h}(v)}:=\hat{h}_{1}(v, h), \end{aligned}$$

19c $$\begin{aligned} \frac{d c_{\mathrm{tot}}}{d\tau} =&R_{c_{\mathrm{tot}}} \biggl(-\frac {1}{K_{c_{\mathrm{tot}}}} \tilde{I}_{\mathrm{Ca}}- \bar{\phi}(ca_{i}) \frac {Ca_{i}}{Q_{c}} \biggr):=\hat{g}(v, ca_{i}), \end{aligned}$$

19d $$\begin{aligned} \varepsilon\frac{dca_{i}}{d\tau} =& (\bar{J}_{\mathrm{ER}_{\mathrm{IN}}}-\bar {J}_{\mathrm{ER}_{\mathrm{OUT}}} )+ \biggl(-\hat{I}_{\mathrm{Ca}}- \bar {K}_{c_{\mathrm{tot}}}\bar{ \phi}(ca_{i}) \frac{Ca_{i}}{Q_{c}} \biggr) \\ :=&\hat{f}_{2}(v, ca_{i}, c_{\mathrm{tot}},l), \end{aligned}$$

19e $$\begin{aligned} \frac{dl}{d\tau} =& R_{l} \bigl(\bar{K}_{\mathrm{d}}(1-l)-ca_{i} l\bigr):=\hat {g}_{3}(ca_{i}, l), \end{aligned}$$

with small parameter *ε*, where $R_{h}$, $R_{c_{\mathrm{tot}}}$, and $R_{l}$ are dimensionless parameters bounded by one. This is not yet the system (7a)–(7e) given in Sect. 4. To obtain that, we notice that $\mathrm{Ca}_{\mathrm{i}}$ values vary significantly across different phases of the solution, which will affect the timescale of $c_{\mathrm{tot}}$. Specifically, by numerical simulations, we have $\mathrm{Ca}_{\mathrm{i}}=O(0.01)~\mu\mbox{M}$ (i.e., $\frac{\mathrm{Ca}_{\mathrm{i}}}{Q_{c}}=O(0.01)$) during the silent phase of the SS solution and $\mathrm{Ca}_{\mathrm{i}}= 1~\mu\mbox{M}$ (i.e., $\frac{\mathrm{Ca}_{\mathrm{i}}}{Q_{c}}=O(1)$) during the active phase. We have $1/K_{c_{\mathrm{tot}}}=O(0.01)$ in (19c). Set

20a $$\begin{aligned} \delta :=&1/K_{c_{\mathrm{tot}}}, \qquad -R_{c_{\mathrm{tot}}}\tilde{I}_{\mathrm{Ca}}:= \hat {g}_{1}(v), \end{aligned}$$

20b $$\begin{aligned} d :=&K_{c_{\mathrm{tot}}} \frac{\mathrm{Ca}_{\mathrm{i}}}{Q_{C}},\qquad -R_{c_{\mathrm{tot}}}\bar{\phi }(ca_{i}):=\hat{g}_{2}(ca_{i}), \end{aligned}$$

such that we can write

21 $$\begin{aligned} \hat{g}(v,ca_{i})=\delta\hat{g}_{1}(v)+d \delta\hat{g}_{2}(ca_{i}), \end{aligned}$$

with small parameter *δ*, where $\hat{g}_{1}$ and $\hat{g}_{2}$ are bounded by one. The value *d* is determined by $\frac{\mathrm{Ca}_{\mathrm{i}}}{Q_{c}}$, which has an $O(1)$ size during the silent phase and a size that is approximately $1/\delta$ during the active phase. Recall that we have picked the slow time $t_{s}$ as our reference time, hence $\tau=t_{s}$ in the above system. As a result, the dimensionless system (19a)–(19e) with $g(v, ca_{i})$ given in (21) becomes the system (7a)–(7e) in Sect. 4, which features three distinct timescales during the silent phase and, due to the larger size of *d*, two timescales during the active phase.
